# Quzhou Fructus Aurantii Extract suppresses inflammation via regulation of MAPK, NF-κB, and AMPK signaling pathway

**DOI:** 10.1038/s41598-020-58566-7

**Published:** 2020-01-31

**Authors:** Lili Li, Jiaoting Chen, Lin Lin, Guixuan Pan, Sheng Zhang, Hao Chen, Majuan Zhang, Yaoxian Xuan, Yin Wang, Zhenqiang You

**Affiliations:** 1 Zhejiang Academy of Medical Sciences, Hangzhou Medical College, Hangzhou, Zhejiang China; 2 Collaborative Innovation Center of Yangtze River Delta Region Green Pharmaceuticals, Zhejiang University of Technology, Hangzhou, Zhejiang China

**Keywords:** Pharmacodynamics, Toxicology

## Abstract

The anti-inflammatory activity of Quzhou Fructus Aurantii Extract (QFAE) has been reported recently. Thus, present study aims to explore the mechanism of anti-inflammation of QFAE *in vitro* and *in vivo* to develop a lung phylactic agent. The anti-inflammatory mechanism of QFAE in RAW 264.7 cells and acute lung injury (ALI) mice model was determined by cytokines analysis, histopathological examination, Western blot assay, immunofluorescence, and immunohistochemistry analysis. The results showed that QFAE restrained mitogen-activated protein kinase (MAPK) and nuclear factor-kappa B (NF-κB) signaling pathways in LPS-induced RAW 264.7 cells, whereas AMP-activated protein kinase (AMPK) signaling pathways were activated, as revealed by prominent attenuation of phosphorylation of ERK, JNK, p38, p65, IκBα, RSK and MSK, and overt enhancement of phosphorylation of ACC and AMPKα. The levels of pro-inflammatory cytokines TNF, IL-6, and IL-1β were suppressed, whereas the level of anti-inflammatory cytokine IL-10 increased after pretreatment with QFAE *in vivo* and *in vitro*. Moreover, QFAE prevented mice from LPS-provoked ALI, bases on alleviating neutrophils, and macrophages in bronchoalveolar lavage fluid (BALF) and mitigatingpulmonary histological alters, as well as hematological change. The MAPK and NF-κB signaling pathways in LPS-stimulated ALI mice were dampened by QFAE pretreatment, whereas AMPK signaling pathways were accelerated, as testify by significant restraint of phosphorylation of ERK, JNK, p38, p65, and IκBα, and distinct elevation of phosphorylation of ACC and AMPKα. The remarkable anti-inflammatory effect of QFAE is associated with the suppression of MAPK and NF-κB signaling pathways and the initiation of AMPK signaling pathway.

## Introduction

Acute lung injury (ALI) and its severe manifestation, acute respiratory distress syndrome (ARDS) have been acknowledged as rapid-onset and life-threatening diseases that can be found in patients of all ages^1–3^. The current clinical difficulty is the present high rates of morbidity and mortality due to ALI/ARDS and lack of proper therapeutic medicine to improve survival^4–6^.

Lipopolysaccharide (LPS) is a commonly used endotoxin for the induction of ALI and can reproduce the major features of acute respiratory inflammatory response and even mortality *in vivo*^7,8^. On the basis of the previous studies of other research groups, mitogen-activated protein kinase (MAPK), nuclear factor-kappa B (NF-κB) and AMP-activated protein kinase (AMPK) signaling pathways are major signaling pathways associated with LPS-induced lung injury. Several studies showed that LPS is a potent stimulator that triggers MAPK and NF-κB signaling pathways and suppresses AMPK signaling pathway^9–11^. The MAPKs, including ERK1/2, JNK, p38, and ERK5 four subfamilies, are a family of serine/threonine protein kinases that regulate fundamental biological processes and cellular responses to external stress signals, can be activated by various pro-inflammatory stimuli, and play a crucial role in the pathogenesis and development of inflammation^12,13^. The NF-κB, an important transcriptional regulation factor in the cells, has been considered as a representative pro-inflammatory signaling pathway owing to its role in the expression of pro-inflammatory genes, including cytokines, chemokines, and adhesion molecules^14,15^. The AMPK is an extremely conservative protein kinase, and exists in all eukaryotic cells in the form of heterotrimeric complexes that functions as a key regulator of metabolism, and expresses remarkable anti-inflammatory and immunosuppressive effects in various cell types and models of inflammatory or autoimmune diseases^16,17^.

Quzhou Fructus Aurantii (QFA), recorded in the “Zhejiang Traditional Chinese Medicine Processing Norms (2015)”, was selected in the new “Zhejiang 8 Famous Kinds Herbal Medicines” recently. The QFA is an unripe fruit of *Rutaceae Citrus changshan-huyou Y.B. Chang*inChangshan County, Quzhou City, Zhejiang Province. According to the application of QFA in folk using for treating cough, we raised the viewpoint that QFA could attenuate ALI and even other respiratory inflammation. In our previous research^18^, the role of QFA extract (QFAE) in preventing and treating ALI and inflammation was validated *in vivo* and *in vitro*. The findings exhibited an excellent effect of QFAE on respiratory protection, which may serve as a potential medicine to ALI/ARDS and even other respiratory inflammation cases. Nevertheless, the in-depth molecular mechanisms of anti-inflammation of QFAE remain obscured. In the present study, we explored the underlying molecular mechanisms of QFAE on anti-respiratory inflammation via LPS-induced lung injury to create a foundation for future clinical therapy.

## Methods

### Ethics statement

The study design and protocols were approved by the Ethical Committee of Zhejiang Academy of Medical Sciences (No. 2018-031). All the experiments were conducted in accordance with relevant guidelines and regulations.

### Reagents

Eriocitrin, narirutin, naringin, neohesperidin, hesperidin, and synephrine were purchased from the National Institutes for Food and Drug Control (Beijing, China). LPS and 3-(4,5-dimethyl-thiazol-2-yl)-2,5-diphenyltetrazolium bromide (MTT)were purchased from Sigma-Aldrich (St. Louis, MO, USA). Antibodies against ERK, p-ERK, JNK, p-JNK, p38, p-p38, p-AMPKα, ACC, p-ACC, p65, p-p65, RSK, p-RSK, MSK, p-MSK, α-Tubulin and β-actin were purchased from Cell Signaling Technology (Danvers, MA, USA). Antibodies against AMPKα was purchased from R&D Systems (Minneapolis, MN, USA). Antibodies against IκBα and p-IκBα were purchased from Santa Cruz Biotechnology (Dallas, Texas, USA). Antibodies against MPO was purchased from abcam (Cambridge, MA, USA). Horseradish peroxidase-conjugated secondary antibody was purchased from Hangzhou Baoke Biotechnology. Co., Ltd. (Hangzhou, China). Alexa Fluor 488-conjugated AffiniPure donkey anti-rabbit IgG was purchased from Jackson ImmunoResearch Laboratories, Inc. (West Grove, PA, USA). Dexamethasone (Dex) was purchased from North China Pharmaceutical Qinhuangdao Co., Ltd. (Hebei, China). Mouse Inflammation Kit and IL-1β enzyme-linked immunosorbent assay (ELISA) Kit were purchased from BDBiosciences (San Jose, CA, USA). Enhanced BCA Protein Assay Kit and Enhanced Chemiluminescence (ECL) Kit were purchased from Beyotime Biotechnology (Shanghai, China).

### Preparation of QFAE

The QFAE preparation was conducted as previously described^18^.

### High-performance liquid chromatography analysis of flavonoids and alkaloid in QFAE

High-performance liquid chromatography (HPLC; Agilent Technologies 1290 Infinity, USA) was utilized for the quantitative determination of various ingredients, such as eriocitrin, narirutin, naringin, neohesperidin, hesperidin, and synephrine in QFAE. Eriocitrin, narirutin, naringin, neohesperidin, and hesperidin were separated on an Eclipse XDB-C18 column (150 mm × 4.60 mm, 5 μm, Agilent) with acetonitrile-0.1% phosphoric acid (volume ratio of 20:80) as the mobile phase. The UV detection wavelength was set at 283 nm; the flow rate was 1.0 mL/min; the column temperature was set at 25 °C; and the injection volume was 10 μL. Synephrine was separated on a Synergi 4 μ Hydre-RP 80 A column (250 mm × 4.60 mm, 4 micro, Phenomenex, USA) with acetonitrile-0.1% phosphoric acid (0.1% sodium dodecyl sulfate) aqueous solution (volume ratio of 32:68) as the mobile phase. The UV detection wavelength was set at 224 nm; the flow rate was 1.0 mL/min; the column temperature was set at 25 °C; and the injection volume was 10 μL.

### Cell culture

A murine macrophage cell line RAW 264.7 was purchased from Shanghai Gefan Biotechnology. Co., Ltd., China. The methods of cell culture were followed as previously described^18^.

### Cell viability assay

The *in vitro* cell viability of QFAE on RAW 264.7 cells was evaluated by MTT assay. Briefly, the cells were plated at a density of 1 × 10^4^ cells/well in 96-well plates. After a 24 h incubation, the cells were treated with QFAE with 0–100 mg/mL dose and cultured for another 24 h. Then, the cells were treated with MTT (5 mg/mL) and incubated for 4 h. Next, the supernatant was removed, and the formazan was resolved in DMSO. The optical density was measured at 570 nm on a microplate reader (Biotek, USA).

### Western blot analysis and cytokine detection of cell

The *in vitro* Western blot analysis consists of two sections, one is to investigate the effect of QFAE on cell itself and the other is to investigate the effect of QFAE on LPS-induced cell inflammation.

RAW 264.7 cells were seeded in 6-well plates at a density of 1 × 10^5^ cells/well and incubated for 24 h. Then, the cells were treated with 25, 10, 5, and 1 mg/mL of QFAE. Simultaneously, the blank control group was set. The cells were incubated for another 24 h. Subsequently, the supernatant was removed, and the cell samples were prepared for Western blot analysis.

RAW 264.7 cells were seeded in 6-well plates at a density of 1 × 10^6^ cells/well and incubated for 24 h. Then, the cells were treated with 25, 10, 5, and 1 mg/mL of QFAE for 1 h before LPS (1 μg/mL) stimulation. Simultaneously, the blank control, drug control (QFAE 25 mg/mL), and model control (LPS 1 μg/mL) groups were set. The cells were incubated for another 24 h. Subsequently, the cell-free supernatant was collected for later analysis of cytokine levels of TNF-α, IL-6, and IL-10, which were analyzed following the manufacturer’s instructions by Mouse Inflammation Kit using a flow cytometer (BD FACSCalibur™ Flow Cytometer, USA). The IL-1β was analyzed by using a special ELISA kit according to the manufacturer’s instructions. Then, the cell samples were prepared for Western blot analysis.

The steps of Western blot were followed as previously described^18^.

### Immunofluorescence assay

RAW 264.7 cells were cultured in 24-well plates with glass slides at 1 × 10^5^ cells/well. Cells were incubated in the presence of QFAE (25 mg/mL) for 1 h before LPS stimulation (1 μg/mL). Simultaneously, the blank control, drug control (QFAE 25 mg/mL), and model control (LPS 1 μg/mL) groups were set. After 24 h, the cells were washed with cold PBS, fixed with 4% formaldehyde, permeabilized with 0.5% Triton X-100, and then blocked with 2% BSA. Subsequently, the cells were incubated overnight at 4 °C in a humid chamber with p65 antibodies (1:400), followed by incubation with Alexa Fluor 488-conjugated Affini Pure donkey anti-rabbit IgG (Jackson Immuno Research Laboratories, Inc., USA) for 30 min at room temperature in the dark. The nuclei were visualized by 4′,6-diamidino-2-phenylindole (DAPI) staining for 5 min. The immunofluorescence results were observed under a fluorescence microscope.

### Animals and treatment

A total of 60 male specific pathogen-free ICR mice weighing approximately 18–22 g (6–8 weeks old) were obtained from Shanghai Slack Laboratory Animal Co., Ltd. The animal feeding was followed as previously described^18^.

The mice were randomly divided into six groups (n = 10 each): blank control group (distilled water 21 mL/kg), drug control group (QFAE 21 g/kg), model control group (LPS), treatment groups (QFAE 7 g/kg + LPS, QFAE 21 g/kg + LPS), and positive control group (Dex 5 mg/kg + LPS). On days 0–2, QFAE was administered intragastrically to the drug control and treatment groupsat the indicated doses. The mice in the blank control and model control groups received distilled water. The positive group received Dexamethasone (Dex) intraperitoneally only on day 2. Approximately 1 h after administration on day 2, all the mice (excluding the blank control and drug control groups) were given light anesthesia via intraperitoneal injection with 0.5% pentobarbital sodium and intranasal administration with 20 μg of LPS in 50 μL of sterile saline to induce acute lung injury. The mice in the blank control and drug control groups received 50 µL of sterile saline. On day 3, the mice were sacrificed and blood samples were collected, and the whole lungs of 5 mice in each group were used to collect bronchoalveolar lavage fluid (BALF), the left lungs of other 5 mice were sliced for histopathological examination and immunohistochemistry analysis, and the right lungs of which were used for Western blot analysis.

### Blood and BALF analysis

The acquisition and analysis of blood and BALF were followed as previously described^18^. The cell-free supernatant of BALF was used for the analysis of the cytokine levels (TNF-α, IL-6, and IL-10) following the manufacturer’s instructions by Mouse Inflammation Kit using a flow cytometer. The production of cytokine level of IL-1β was analyzed with a special ELISA kit according to the manufacturer’s instructions.

### Histopathological examination

The histopathological examination was followed as previously described^18^. The severity of histological changes in the lungs was scored semiquantitatively from 0 to 4 as previously described^19^.

### Immunohistochemistry analysis

The immunohistochemistry analysis was followed as previously described^18^.

### Western blot analysis of lung tissues

The steps of Western blot were followed as previously described^18^.

### Statistical analysis

All data were expressed as mean ± standard error of mean (SEM) and analyzed with SPSS 22.0. Statistical comparisons between the groups were conducted by one-way ANOVA. Statistical significance was defined as *P* < 0.05.

## Results

### Component analysis and cytotoxicity of QFAE

**Figure 1:**
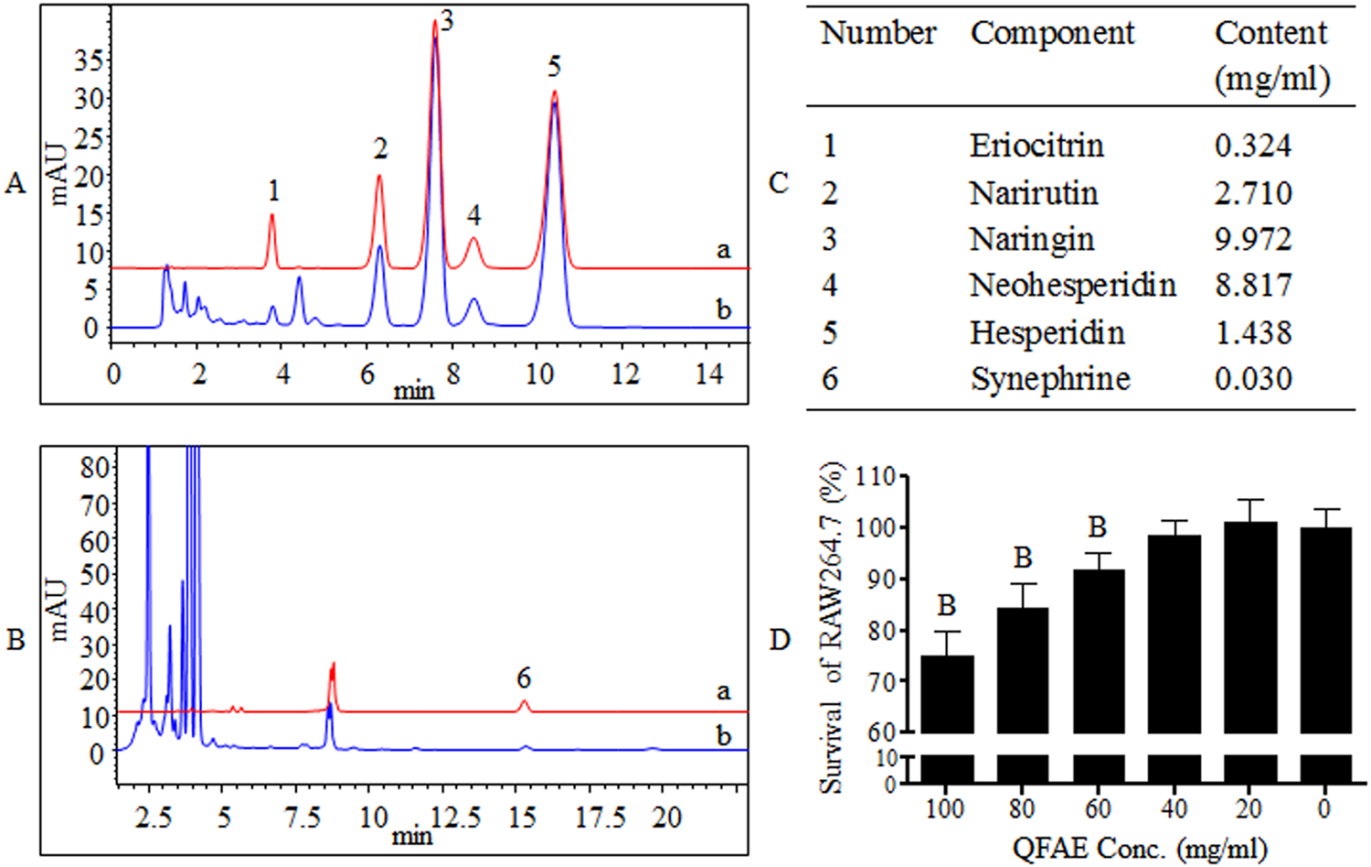
Component analysis and cytotoxicity of QFAE. **(A)** HPLC chromatogram obtained at 283 nm from (a) standard products of eriocitrin, narirutin, naringin, neohesperidin, and hesperidin, (b) QFAE. **(B)** HPLC chromatogram obtained at 224 nm from (a) standard product of synephrine, (b) QFAE. **(C)** Contents of eriocitrin, narirutin, naringin, neohesperidin, hesperidin, andsynephrine in QFAE. **(D)** Effect of QFAE on cytotoxicity in RAW 264.7 cells was determined by MTT assay.

The HPLC was applied for the quantitative determination of ingredients in QFAE, including eriocitrin, narirutin, naringin, neohesperidin, hesperidin, and synephrine. The contents of eriocitrin, narirutin, naringin, neohesperidin, hesperidin, and synephrine in QFAE were 0.324, 2.710, 9.972, 8.817, 1.438, and 0.030 mg/mL, respectively (Fig. 1A–C). The MTT assay was conducted to evaluate the potential cytotoxicity of QFAE on RAW 264.7 cells. The results showed that QFAE was unable to affect the viability of cells at various concentrations from 0 to 40 mg/mL (Fig. 1D).

### QFAE regulates MAPK signaling pathways in RAW 264.7 cells

**Figure 2:**
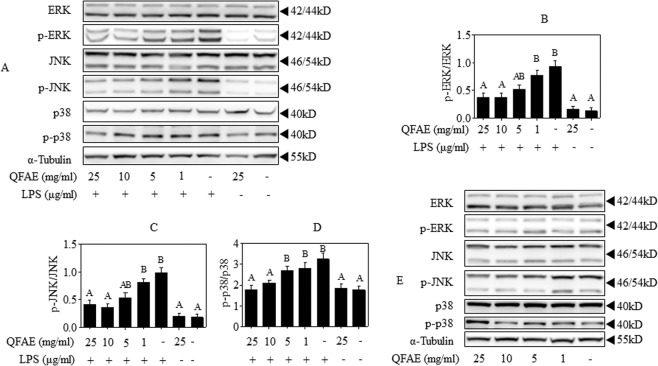
QFAE regulates MAPK signaling pathways in RAW 264.7 cells. **(A)** Protein expressions of total and phosphorylated ERK, JNK, and p38 in LPS-induced RAW 264.7 cells were measured by Western blot, and α-Tubulin was used as a loading control. **(B–D)** Relative protein expressions of p-ERK/ERK, p-JNK/JNK, and p-p38/p38 were quantified by Image J software. **(E)** Protein expressions of total and phosphorylated ERK, JNK, and p38 in none-induced RAW 264.7 cells were measured by Western blot, and α-Tubulin was used as a loading control. Data are presented as the means ± SEM, n = 5. ^*A*^*P* < 0.05 compared with model control group; ^*B*^*P* < 0.05 compared with blank control group.

The effect of anti-inflammation of QFAE on MAPK signaling pathways was examined in LPS-induced RAW 264.7 cells to illustrate its potential mechanisms. Western blot results showed that LPS significantly accelerated the phosphorylation of ERK, JNK, and p38, which was prominently attenuated by QFAE pretreatment (Fig. 2A–D). Furthermore, the influence of QFAE on MAPK signaling pathways was investigated when administered to RAW 264.7 cells alone. Western blot results showed that QFAE had no significant effect on the phosphorylation of ERK, JNK, and p38, thereby indicating that QFAE had no effect on MAPK signaling pathways when administered to RAW 264.7 cells alone (Fig. 2E). The abovementioned findings reveal that the anti-inflammatory effect of QFAE in LPS-induced RAW 264.7 cells is related to MAPK signaling pathways.

### QFAE adjusts AMPK signaling pathways in RAW 264.7 cells

**Figure 3:**
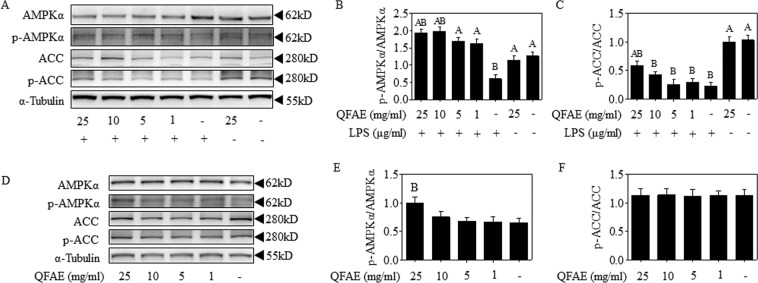
QFAE adjusts AMPK signaling pathways in RAW 264.7 cells. **(A)** Protein expressions of total and phosphorylated AMPKα and ACC in LPS-induced RAW 264.7 cells were measured by Western blot, and α-Tubulin was used as a loading control. **(B**,**C)** Relative protein expressions of p-AMPKα/AMPKα and p-ACC/ACC in LPS-induced RAW 264.7 cells. **(D)** Protein expressions of total and phosphorylated AMPKα and ACC in none-induced RAW 264.7 cells were measured by Western blot, and α-Tubulin was used as a loading control. **(E**,**F)** Relative protein expressions of p-AMPKα/AMPKα and p-ACC/ACC in none-induced RAW 264.7 cells. Data are presented as the means ± SEM, n = 5. ^*A*^*P* < 0.05 compared with model control group; ^*B*^*P* < 0.05 compared with blank control group.

Next, the effect of QFAE on AMPK signaling pathways was analyzed in LPS-induced RAW 264.7 cells. Western blot results discovered that the phosphorylation of AMPKα and ACC was overtly inhibited due to exposure to LPS, which was evidently enhanced by QFAE pretreatment, as shown in Fig. 3A–C. In addition, the alteration of QFAE on AMPK signaling pathways was detected when administered to RAW 264.7 cells alone (Fig. 3D–F). The phosphorylation of AMPKα was significantly increased with 25 mg/mL concentration, thereby indicating that AMPK signaling pathway might be activated when QFAE acted on RAW 264.7 cells alone. These findings prove that the anti-inflammatory effect of QFAE in LPS-induced RAW 264.7 cells involves AMPK signaling pathways.

### QFAE coordinates NF-κB signaling pathways in RAW 264.7 cells

**Figure 4:**
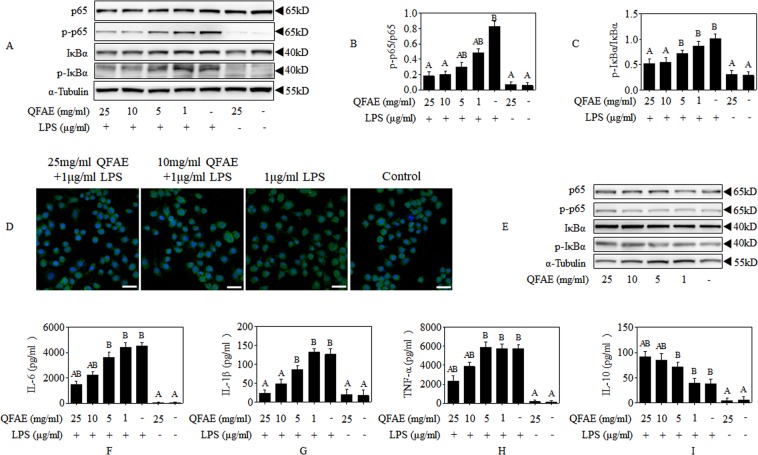
QFAE coordinates NF-κB signaling pathways in RAW 264.7 cells. **(A)** Protein expressions of total and phosphorylated p65 and IκBα in LPS-induced RAW 264.7 cells were measured by Western blot, and α-Tubulin was used as a loading control. **(B**,**C)** Relative protein expressions of p-p65/p65 and p-IκBα/IκBα in LPS-induced RAW 264.7 cells. **(D)** NF-κB subunit p65 localization was verified by immunofluorescence staining with p65 antibody (400 × magnification, 50 μm scale bar). **(E)** Protein expressions of total and phosphorylated p65 and IκBα in none-induced RAW 264.7 cells were measured by Western blot, and α-Tubulin was used as a loading control. **(F**–**I)** Effect of QFAE on cytokines of IL-6, IL-1β, TNF-α, and IL-10 in cell-free supernatant of LPS-induced RAW 264.7 cells. Data are presented as the means ± SEM, n = 5. ^*A*^*P* < 0.05 compared with model control group; ^*B*^*P* < 0.05 compared with blank control group.

NF-κB signaling pathways play a crucial role in LPS-stimulated relevant models. As shown in Fig. 4A–C, the phosphorylation of p65 and IκBα were saliently increased following exposure to LPS alone, which was dramatically reversed by QFAE pretreatment. Moreover, immunofluorescence staining of p65 nuclear translocation demonstrated that pretreatment of QFAE conspicuously suppressed the LPS-induced p65 nuclear translocation, as shown in Fig. 4D. The effect of QFAE on NF-κB signaling pathways when administered to RAW 264.7 cells alone was also observed. The results shown in Fig. 4E illustrated that QFAE had no apparent effect on the phosphorylation of p65 and IκBα, thereby indicating no effect was observed on NF-κB signaling pathways when QFAE was administered to RAW 264.7 cells alone.

The generation of cytokines was mainly regulated by NF-κB signaling pathways. In present study, the pro-inflammatory cytokines IL-6, IL-1β, and TNF-α were conspicuously increased by LPS stimulation in RAW 264.7 cells, while the anti-inflammatory cytokine IL-10 were significantly decreased, as shown in Fig. 4F–I. However, pretreatment with QFAE distinctly prevented the elevated pro-inflammatory cytokines and promoted the decreased IL-10.

These results indicated that QFAE could attenuate the production of inflammatory cytokines provoked by LPS via restraint NF-κB signaling pathways.

### QFAE mediates the phosphorylation of RSK and MSK in RAW 264.7 cells

**Figure 5:**
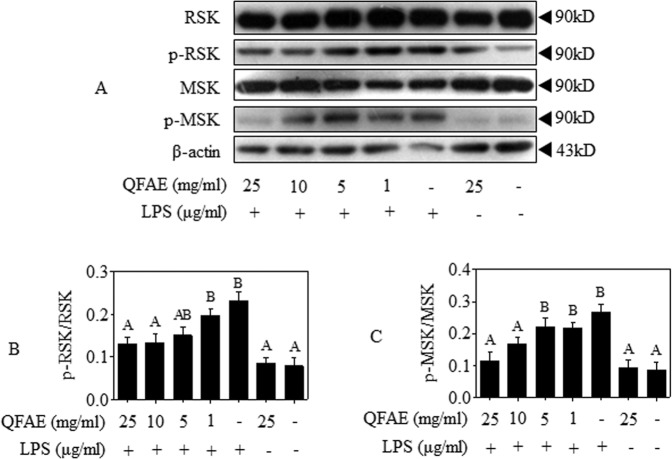
QFAE mediates the phosphorylation of RSK and MSK in RAW 264.7 cells. **(A)** Protein expressions of total and phosphorylated RSK and MSK in LPS-induced RAW 264.7 cells were measured by Western blot, and β-actin was used as a loading control. **(B–C)** Relative protein expressions of p-RSK/RSK and p-MSK/MSK in LPS-induced RAW 264.7 cells. Data are presented as the means ± SEM, n = 5. ^*A*^*P* < 0.05 compared with model control group; ^*B*^*P* < 0.05 compared with blank control group.

The MSK, activated by p38 or ERK via cascade phosphorylation, leads to the transcriptional activation of NF-κB^20,21^. The RSK could be exclusively activated by phosphorylation of ERK in the MAPK signaling pathway and then participates in NF-κB activity^22,23^. The activities of RSK and MSK were examined after LPS induction. Western blot results showed that the phosphorylation of RSK and MSK was increased by LPS exposure but remarkably suppressed by pretreatment with QFAE (Fig. 5A–C).

### QFAE protects against LPS-induced lung injury and inflammation in mice

**Figure 6:**
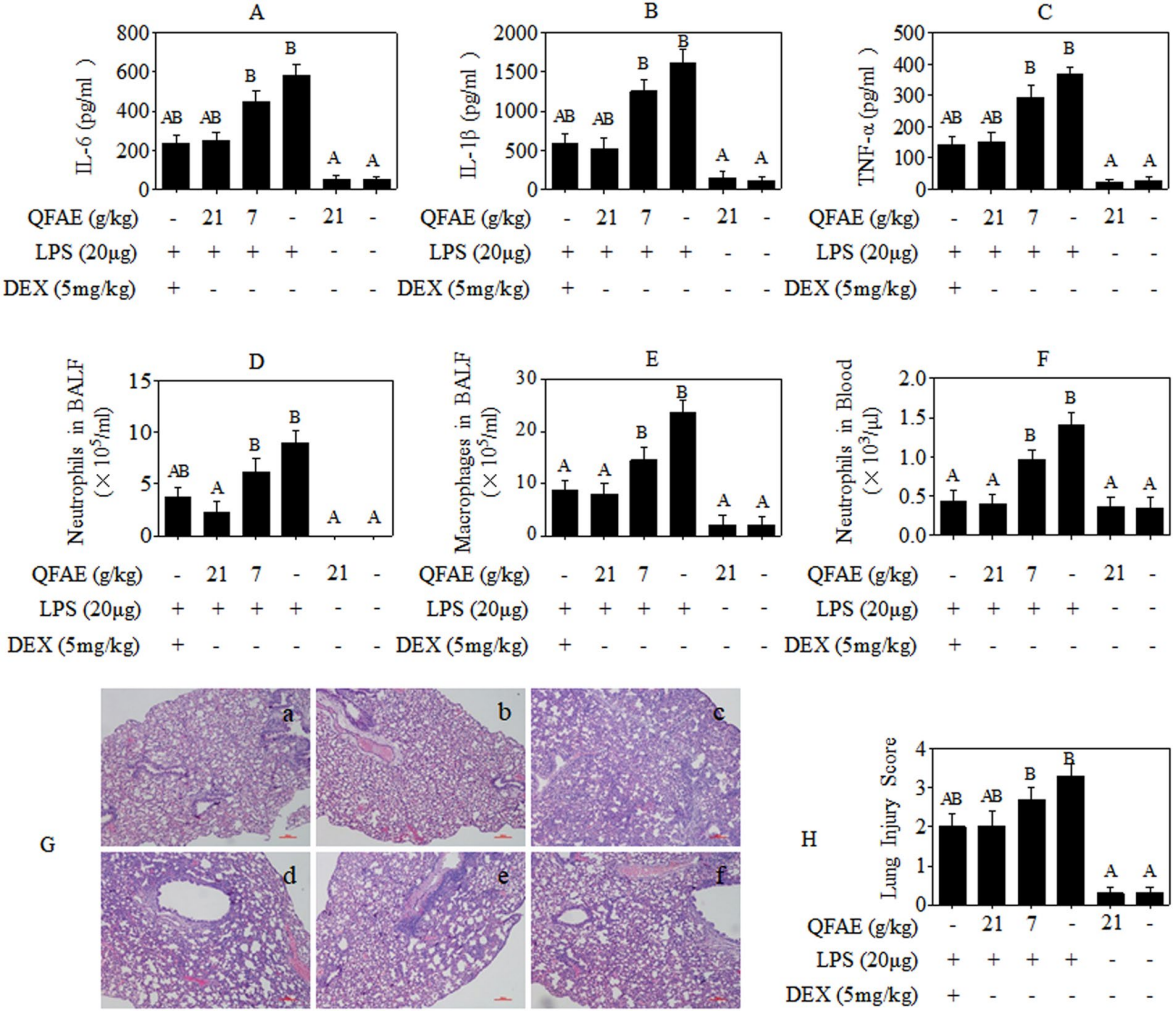
QFAE attenuated LPS-induced lung injury and inflammation in LPS-induced ALI mice. **(A–C)** Cytokine levels of IL-6, IL-1β, and TNF-α in BALF supernatant from LPS-induced ALI mice. **(D**,**E)** Levels of neutrophils and macrophages in BALF from LPS-induced ALI mice. **(F)** Level of neutrophils in blood from LPS-induced ALI mice. **(G**,**H)** Histopathological examination and evaluation of lung lesion in LPS-induced ALI mice (100 × magnification, 100 μm scale bar). Data are presented as the means ± SEM, n = 5. ^*A*^*P* < 0.05 compared with model control group; ^*B*^*P* < 0.05 compared with blank control group.

The ability of QFAE to prevent mice from LPS-stimulated acute lung injury was analyzed. First, the levels of inflammatory cytokines in the BALF supernatant from ALI mice were detected. As illustrated in Fig. 6A–D, the levels of pro-inflammatory cytokines IL-6, IL-1β, and TNF-α were markedly increased in BALF with exposure to LPS, while the level of anti-inflammatory cytokine IL-10 was apparently decreased, but a significant reversal by QFAE pretreatment in high dose group. In addition, cell counting and classification in BALF were performed. Neutrophils and macrophages were evidently increased after LPS provocation, but were notably ameliorated by pretreatment with QFAE on the dose of 21 g/kg (Fig. 6D,E). Hematological analysis also indicated that QFAE (21 g/kg) exhibited an arresting influence on decreasing the level of neutrophils enhanced by LPS induction (Fig. 6F). Furthermore, H&E staining was used to determine the degree of lung lesion in LPS-induced ALI mice. The drug control and blank control groups indicated no histopathologic alteration in the lung tissues. Severe pulmonary hemorrhage, evident alveolar collapse, and salient neutrophil infiltration were observed in the model control group. However, QFAE pretreatment (21 g/kg) ameliorated the abovementioned histopathological changes noticeably (Fig. 6G,H). The analogous treatment effects were obtained in the positive control group. The abovementioned findings reveal that QFAE attenuates lung injury in LPS-induced ALI mice and exhibits an excellent anti-inflammatory activity *in vivo*.

### QFAE suppresses MAPK signaling pathways in LPS-induced ALI mice

**Figure 7:**
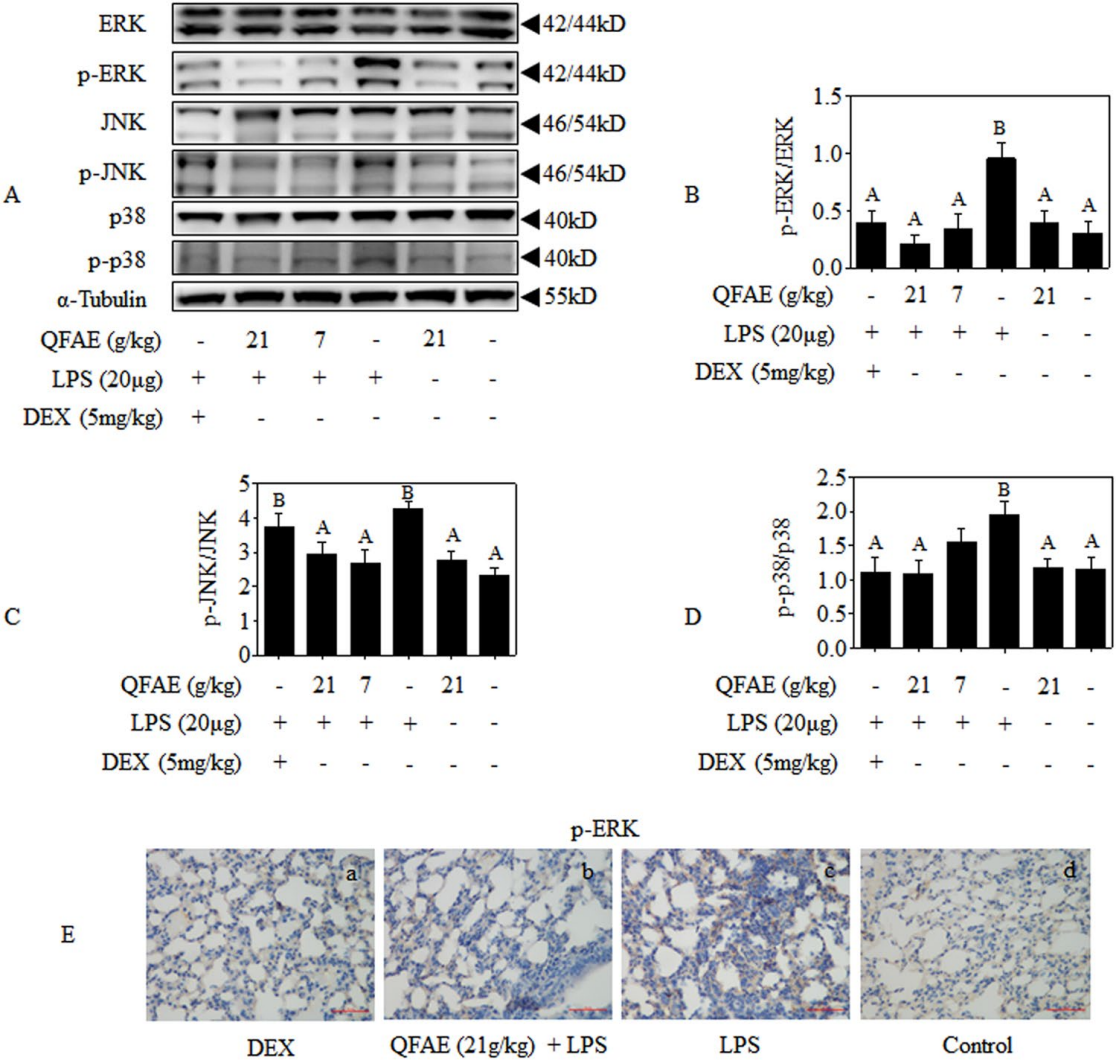
QFAE suppresses MAPK signaling pathways in LPS-induced ALI mice. **(A)** Protein expressions of total and phosphorylated ERK, JNK, and p38 in LPS-induced ALI mice were measured by Western blot, and α-Tubulin was used as a loading control. **(B**–**D)** Relative protein expressions of p-ERK/ERK, p-JNK/JNK, and p-p38/p38 were quantified by Image J software. **(E)** Expression of p-ERK was determined by immunohistochemistry (400 × magnification, 50 μm scale bar). Data are presented as the means ± SEM, n = 5. ^*A*^*P* < 0.05 compared with model control group; ^*B*^*P* < 0.05 compared with blank control group.

The effects of the anti-inflammation of QFAE on MAPK signaling pathway in the lung tissues of LPS-induced ALI mice were investigated to further elucidate its underlying mechanisms. As shown in Fig. 7A–D, Western blot results revealed that QFAE pretreatment significantly restrained LPS-induced relative phosphorylation of ERK, JNK, and p38. No apparent effect exists on the expression levels of total ERK, JNK, and p38 after treatment with QFAE and LPS. Similar treatment effects were obtained in the positive control group. Moreover, immunohistochemistry analysis of p-ERK also proved that pretreatment of QFAE prevented the LPS-induced phosphorylation in ALI mice (Fig. 7E). The abovementioned results prove that the anti-inflammatory effect of QFAE in LPS-induced ALI mice is related to MAPK signaling pathways.

### QFAE inhibits NF-κB signaling pathways in LPS-induced ALI mice

**Figure 8:**
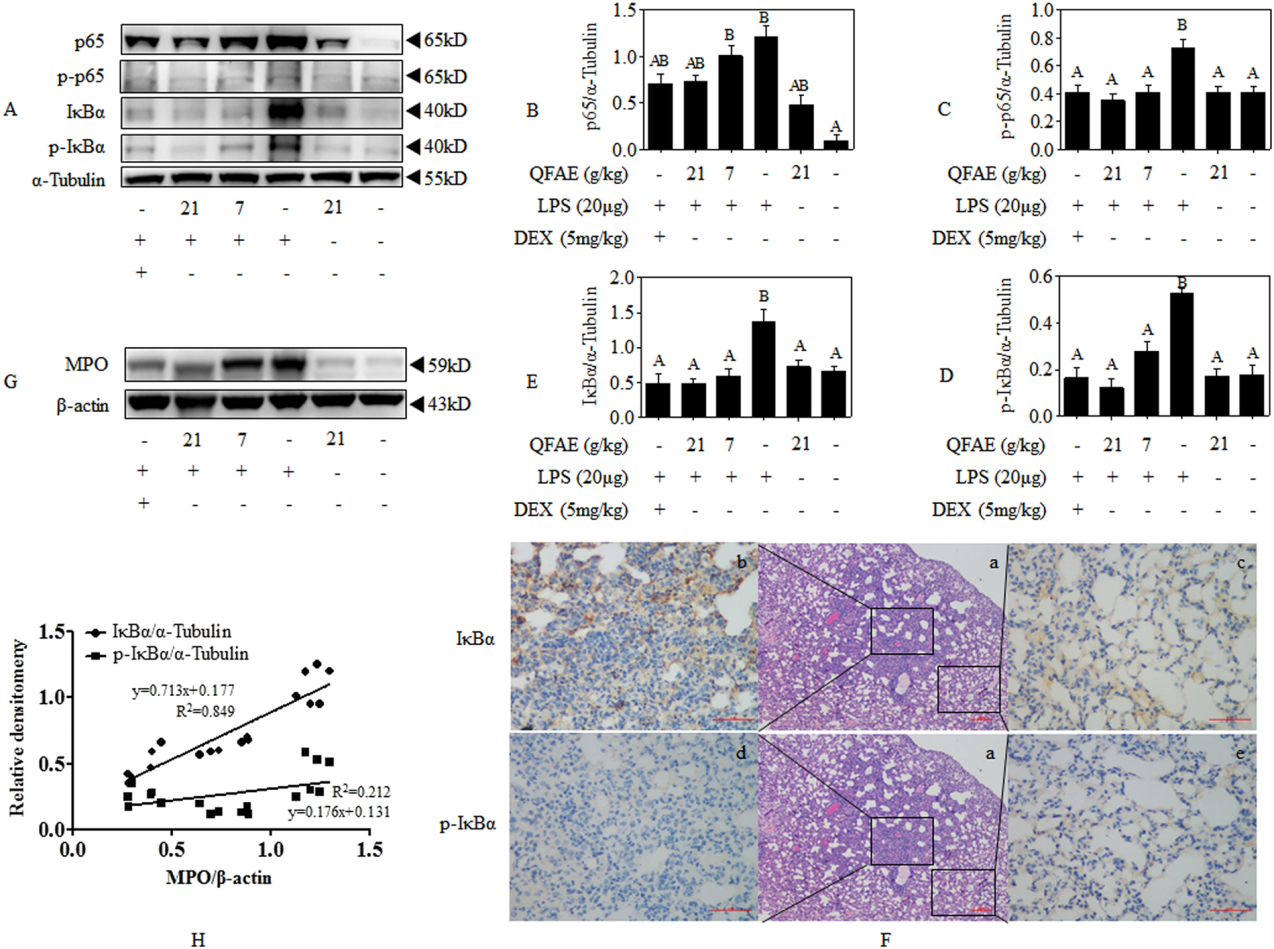
QFAE inhibits NF-κB signaling pathways in LPS-induced ALI mice. **(A)** Protein expressions of total and phosphorylated p65 and IκBα in LPS-induced ALI mice were measured by Western blot, and α-Tubulin was used as a loading control. **(B–E)** Relative protein expressions of p65, p-p65, IκBα, and p-IκBα in LPS-induced ALI mice. **(F)** Expression of IκBα and p-IκBα were determined by immunohistochemistry (400 × magnification, 50 μm scale bar). (**G**) Protein expressions of MPO in LPS-induced ALI mice were measured by Western blot, and β-actin was used as a loading control. (**H**) Correlation analysis between IκBα, p-IκBα, and MPO. Data are presented as the means ± SEM, n = 5. ^*A*^*P* < 0.05 compared with model control group; ^*B*^*P* < 0.05 compared with blank control group.

As illustrated in Fig. 8A–E, Western blot results indicated that the relative expressions of p65, p-p65, IκBα, and p-IκBα were dramatically elevated compared with that of loading control α-Tubulin following the exposure to LPS alone in the lung tissues of ALI mice, whereas prominently reversed by pretreatment with QFAE. In addition, immunohistochemistry results indicated that the expression level of IκBα was increased in the inflammatory regions of the lung tissue in the model control group, while the expression level of which in the comparatively normal regions was particularly low. However, no significant difference was observed in the expression of p-IκBα between the inflammatory regions and the normal regions in the model control group (Fig. 8F). On this basis, correlation statistics were used to analyze the correlation between IκBα, p-IκBα, and MPO. The results showed that IκBα/α-Tubulin was conspicuously correlated with MPO/β-actin, but the association of p-IκBα/α-Tubulin and MPO/β-actin was at the low level. Thus, no significant difference was observed between the expression of IκBα and MPO, and a marked difference exists between the expression of p-IκBα and MPO (Fig. 8G,H). Simultaneously, p65 and p-p65 obtain similar results. Therefore, IκBα/MPO, p65/MPO, p-p65/α-Tubulin, and p-IκBα/α-Tubulin were adopted to comprehensively analyze the relative phosphorylation, thereby indicating that QFAE regulates NF-κB signaling pathways by inhibiting the expression levels of p-IκBα and p-p65 caused by LPS in ALI mice.

### QFAE activates AMPK signaling pathways in LPS-induced ALI mice

**Figure 9:**
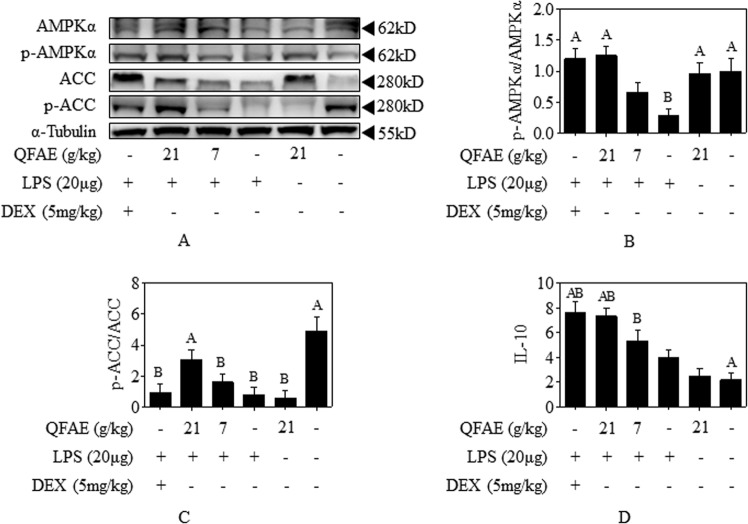
QFAE activates AMPK signaling pathways in LPS-induced ALI mice. **(A)** Protein expressions of total and phosphorylated AMPKα and ACC in LPS-induced ALI mice were measured by Western blot, and α-Tubulin was used as a loading control. **(B**,**C)** Relative protein expressions of p-AMPKα/AMPKα and p-ACC/ACC in LPS-induced ALI mice. **(D)** Cytokine level of IL-10 in BALF supernatant from LPS-induced ALI mice. Data are presented as the means ± SEM, n = 5. ^*A*^*P* < 0.05 compared with model control group; ^*B*^*P* < 0.05 compared with blank control group.

The effect of QFAE on AMPK signaling pathways in LPS-induced ALI mice was analyzed. Western blot results revealed that LPS notably suppressed the relative phosphorylation of AMPKα and ACC in the lung tissue, while pretreatment with QFAE (21 g/kg) distinctly raised its relative phosphorylation, thereby indicating that QFAE exerts an anti-inflammatory role by activating the AMPK signaling pathways (Fig. 9A–C). Similar effects of Dex were revealed in the positive control group.

## Discussion

The QFA, also known as Changshanhuyou traditionally, as a conventional medicine with a long history in Changshan County, Quzhou City, Zhejiang Province, should be harvested in July when the peel is still green. Previous studies have shown that the ingredients of the QFA are mainly consistent with that of Fructus Aurantii^24–27^, primarily including nobiletin, eriocitrin, narirutin, naringin, neohesperidin, hesperidin, and synephrine, which indicated that QFA could be used as a substitute for Fructus Aurantii. In our pre-experiment, we conducted an assay of maximum administration dosage (MAD) of QFAE on ICR mice. The results revealed that MAD was 100 g/kg, and there was no adverse reaction on mice. In another study, SD rats were administered intragastrically with 40 g/kg/d QFAE (equivalent to a mouse dose of 60 g/kg/d), and were administered for 30 days in a row. The results showed that there were no abnormalities in body weight, food intake, hematology, biochemistry and pathology indexes of rats. So the doses of QFAE, used in this study, are safety for mice *in vivo*. Our prior research has detected the content of naringin using as a criterion for the quality control and quantification of QFAE^18^. In present experiment, the components of eriocitrin, narirutin, neohesperidin, hesperidin, and synephrine were additionally tested to provide a basis for comprehensive evaluation of the quality control of QFAE. Moreover, the abovementioned monomers are widely distributed in citrus plants, which have been proven to keep anti-inflammatory and anti-oxidative activities^28–32^. Thus, the anti-inflammatory effects of QFAE may be related to the abovementioned compounds.

The ALI, a kind of damage of alveolar epithelial and capillary endothelial cells caused by various direct or indirect injury factors, is characterized by diffused pulmonary interstitial and alveolar edema, neutrophil accumulation, pulmonary hypertension, pulmonary infiltrates, alveolar rupture, and pulmonary hemorrhage, thereby leading to acute hypoxic respiratory insufficiency and, in severe cases, ARDS^1,6,33^. ALI/ARDS is closely related to sepsis^34,35^, pneumonia^36^ and trauma^37^, with severe incidence and mortality^38^. In our research, LPS was applied for the provocation of ALI in mice to reproduce the major pathological features. Histopathological examination illustrated that massive inflammatory cells aggregated in the lungs of ALI mice along with alveolar rupture and pulmonary hemorrhage, thereby indicating the success of ALI model. The protective effect of QFAE against ALI mice was proven by biochemical and histological examinations, immunohistochemistry, and protein expression analysis.

Cytokines are the key mediators participating in inflammation and host defense, which could be controlled by the post-transcriptional and have relation to activation of multiple signaling cascades during inflammation progression^39^. Pro-inflammatory cytokines are indispensable for immune system, and although they may produce severe inflammatory lesion, tightly regulating their production and excretion is necessary^40,41^. The IL-10 is a potent anti-inflammatory factor in bacterial endotoxemia and crucial for maintaining homeostasis, which could be stimulated by LPS as a negative feedback by dampening a pro-inflammatory response and preventing inflammation caused by tissue injury^42,43^. Our prior and present study showed that the pro-inflammatory cytokines TNF-α, IL-6, and IL-1β exhibited an excess accumulation induced by LPS *in vivo* and *in vitro*, while the anti-inflammatory cytokine IL-10 was an overt abrogation. However, an evident moderation of TNF-α, IL-6, and IL-1β and a prominent elevation of IL-10 were observed by pretreatment with QFAE, thereby indicating that QFAE has an effective anti-inflammatory activity *in vivo* and *in vitro*.

The diversity of intracellular signaling pathways is comprised in the regulation of inflammatory cytokines. MAPK pathway consists of ERK, JNK, and p38 signaling pathways, which could be activated by several pro-inflammatory cytokines (e.g., TNF-α and IL-1β) or in answer to extracellular stress (e.g., oxidative stress and genotoxicity)^44^. NF-κB, an important regulator involved in the regulation of quantity of genes that govern diverse immune and inflammatory responses, is activated by various stimuli that cover oxidative stress, endotoxin, and inflammatory cytokines, can be further enhanced by TNF-α and IL-1β cytokines, and then activates other cells to synthesize various cytokines, such as IL-6 and IL-8, to form a positive feedback response^45,46^. AMPK is a cellular energy regulator and its activation can significantly suppress the levels of pro-inflammatory cytokines, promote the levels of anti-inflammatory cytokines, and exert anti-inflammatory effects, which are mostly accompanied by inhibition of the NF-κB pathways. Meanwhile, its activity is also regulated by cytokines^47–49^. Specifically, the production of pro-inflammatory cytokines is primarily managed by NF-κB signaling pathways and associated with MAPK signaling pathways, and the generation of anti-inflammatory cytokines is correlated with AMPK signaling pathways.

According to the abovementioned results, we focused our attention on MAPK/NF-κB/AMPK signaling pathways that are associated with respiratory inflammation in response to LPS. Previous studies have shown that ERK, JNK, and p38 are closely bound up with various inflammatory diseases, such as acute lung injury, and can be activated by LPS, TNF, and other irritants. When MAPK is activated, phosphorylated ERK, p38, and JNK are transferred to the nucleus alone or in combination to induce expression of relevant inflammatory factor target genes and promote inflammatory response^10,50,51^. Pretreatment of QFAE to LPS induced RAW 264.7 cells dramatically suppressed MAPK signaling pathways as evaluated by the decrease on the phosphorylation of ERK, JNK, and p38. The prevention of QFAE on MAPK signaling pathways was also verified in LPS-stimulated ALI mice by the distinct inhibition of phosphorylation of ERK, JNK, and p38.

AMPK is a key regulator of energy homeostasis. Activation of AMPK directly phosphorylates downstream target ACC, inhibits metabolic pathways, such as fatty acid, cholesterol and protein syntheses, and increases the level of ATP in cells^52^. Although previous studies have confirmed that AMPK has anti-inflammatory effects in lung inflammatory diseases, its specific mechanism remains obscured^53^. In response to various stimulation, the activated AMPK may directly or indirectly regulate the activity of NF-κB through downstream SIRT, Fox 3a, p53, and PGC-1a proteins, thus inhibiting inflammatory factors and playing an anti-inflammatory role^54^. Pretreatment of QFAE to LPS induced RAW 264.7 cells apparently promoted AMPK signaling pathways by upgrading the phosphorylation of AMPK and ACC. The acceleration of QFAE on AMPK signaling pathways was also testified in LPS-stimulated ALI mice by the salient motivation of phosphorylation of AMPK and ACC.

NF-κB exists in the cytoplasm and binds to its inhibitory protein IκB as a trimer form with no transcriptional activity in the resting state. Extracellular stimulation signals are transmitted into the cells via membrane receptors to liberate IκB-inhibited NF-κB and activate it, then rapidly enter the nucleus to specifically bind to the κB sequence of the corresponding target gene regulatory element on DNA and up-regulate the expression of these target genes by individual or synergistic action, thereby exerting relevant physiological or pathological effects. Specifically, when p65 and IκBα complexes are stimulated, IκBα is phosphorylated and then degraded, resulting in p65 dissociation and entry into the nucleus to phosphorylate and induce transcription and expression of inflammatory mediators and immune-related genes^47,48,55,56^. In this study, the phosphorylation of IκBα and p65 was significantly accelerated in LPS stimulated RAW 264.7 cells, thereby indicating the activation of NF-κB signaling pathways, which was remarkably restrained by QFAE treatment.

As previously mentioned, in the analysis of the ability of QFAE to protect ALI mice through NF-κB signaling pathways, pretreatment with QFAE overtly suppressed the relative tubunlin expressions of p65, p-p65, IκBα, and p-IκBα in the lung tissues of ALI mice, whereas the relative phosphorylation, p-p65/p65 and p-IκBα/IκBα, was nonsignificantly different. Furthermore, the immunohistochemistry results in the model control group indicated that the expression of IκBα was increased in the inflammatory areas of the lung tissue, whereas the expression of that in the relatively normal regions was quite low. Moreover, in the model control group, the expression of p-IκBα in the inflammatory and normal regions has no significant difference. These results all indicated the excitation of NF-κB signaling pathways in the lung tissue of ALI mice presented as disassembly and phosphorylation of p65-IκBα complexes due to LPS induction. Additionally, MPO is a particular marker released by neutrophils, macrophages, and monocytes and can reflect the degree of inflammation in the inflammatory process^57,58^. An apparent inhibition was observed on the expression of MPO in the lung tissues of ALI mice pretreated with QFAE. Thus, we proposed a correlation analysis method between IκBα, p-IκBα, and MPO. Canonical correlation analysis is a statistical analysis tool used to identify and quantify the correlation between two sets of variables, which is applied in various fields of research, such as biomedicine, ecology, and genetics^59^. We found the expression of IκBα was prominently correlated with the expression of MPO, but the association of p-IκBα and MPO was particularly low. Similar results of p65 and p-p65 were obtained. The outcomes of immunohistochemistry and correlation analysis indicated that the expressions of IκBα and p65 are closely related to the degree of inflammation, and there was no significant relationship between the expressions of p-IκBα and p-p65 and the degree of inflammation. Therefore, in our study, there was no regular change in the relative phosphorylation of p-p65/p65 and p-IκBα/IκBα, and the relative expressions of p-p65/α-Tubulin and p-IκBα/α-Tubulin were adopted to synthetically evaluate the effect of QFAE in NF-κB signaling pathways in ALI mice.

In addition, RSK and MSK were detected in the present research. The 90 kDa ribosomal S6 kinase (RSK) family of proteins are downstream effectors of ERK/MAPK signaling cascade, which could be directly activated by ERK1/2^22,23,60^. The mitogen- and stress-activated protein kinases (MSKs) that belong to the RSK family of protein kinases are activated by p38 and ERK via cascade phosphorylation, which are regulated by upstream activators and activate downstream targets^20,21,61^. Previous studies have shown that activated RSK and MSK mediated phosphorylation of p65, indicating that RSK and MSK are particular proteins involved in MAPK and NF-κB pathways^62,63^. In this regard, the ability of QFAE to inhibit LPS-induced phosphorylation of RSK and MSK *in vitro* and *in vivo* was investigated. Specifically, QFAE markedly restrained the phosphorylation of RSK and MSK in LPS-stimulated RAW 264.7 cells, as well as the phosphorylation of ERK, p38, and p65. However, our *in vivo* study showed no significant difference in phosphorylation of RSK and MSK, while the phosphorylation of ERK, p38, and p65 were still reversed by pretreatment with QFAE in ALI mice. This condition resulted in a conundrum that needs to be analyzed.

In conclusion, QFAE had an effective anti-inflammation in LPS-induced RAW 264.7 cells and lung phylactic activity in LPS-stimulated ALI mice that saliently alleviated the pro-inflammatory cytokines TNF-α, IL-6, and IL-1β, while noticeably raised the anti-inflammatory cytokine IL-10 *in vitro* and *in vivo*. Besides, QFAE protected against ALI in mice via attenuating pulmonary histological alters and hematological changes. The conspicuously protective effect of QFAE was associated with suppression of MAPK and NF-κB signaling pathways and motivation of AMPK signaling pathways. Therefore, QFAE may serve as a potential lung phylactic agent to ALI/ARDS and even other respiratory inflammation cases.

## Supplementary information

Supplementary Information
