# Supplementary material for: Quzhou Fructus Aurantii Extract suppresses inflammation via regulation of MAPK, NF-κB, and AMPK signaling pathway
Source: Sci Rep. 2020 Jan 31;10:1593. doi: 10.1038/s41598-020-58566-7 (PMC6994495; doi:10.1038/s41598-020-58566-7)

**Quzhou Fructus Aurantii Extract suppresses inflammation via regulation of MAPK, NF-κB, and AMPK signaling pathway**

Lili Li ^1, 2^, Jiaoting Chen ^1, 2^, Lin lin^1^, Guixuan Pan^1^, Sheng Zhang ^1^, Hao Chen ^1^, Majuan Zhang^1^, Yaoxian Xuan^1^, Yin Wang ^1^*, Zhenqiang You ^1^*

*^1^* Zhejiang Academy of Medical Sciences, Hangzhou Medical College, Hangzhou, Zhejiang, China

*^2^*Collaborative Innovation Center of Yangtze River Delta Region Green Pharmaceuticals, Zhejiang University of Technology, Hangzhou, Zhejiang, China

Running title: Quzhou Fructus Aurantii Extract suppresses inflammation

* Correspondence

Zhenqiang You, [youzhenqiang0817@163.com](mailto:youzq1979@163.com)

Yin Wang, [wy3333@163.com](mailto:wy3333@163.com)

Zhejiang Academy of Medical Sciences, Hangzhou Medical College, Hangzhou, Zhejiang, China

**Original images of western blot**

Figure 2A ERK Figure 2A p-ERK


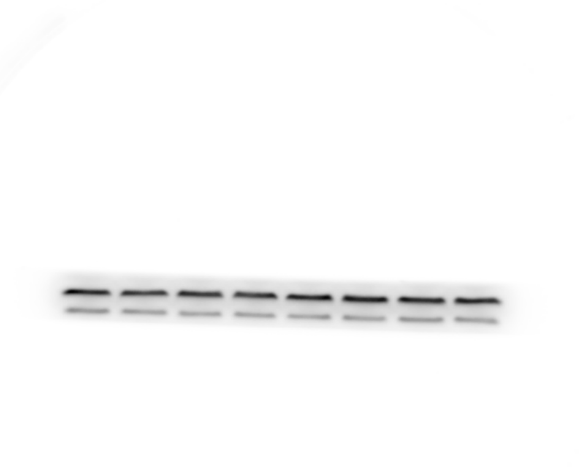

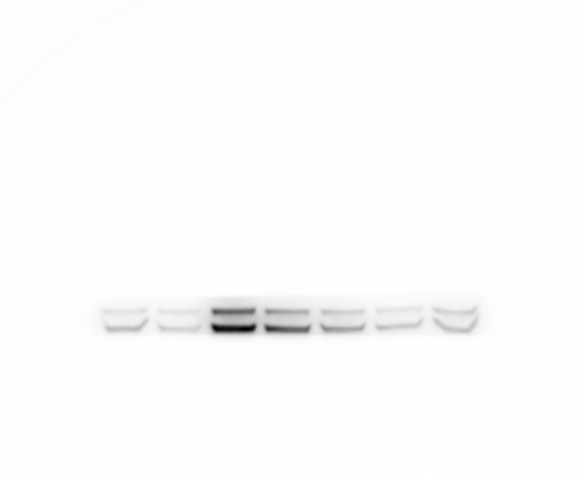


Figure 2A JNK Figure 2A p-JNK


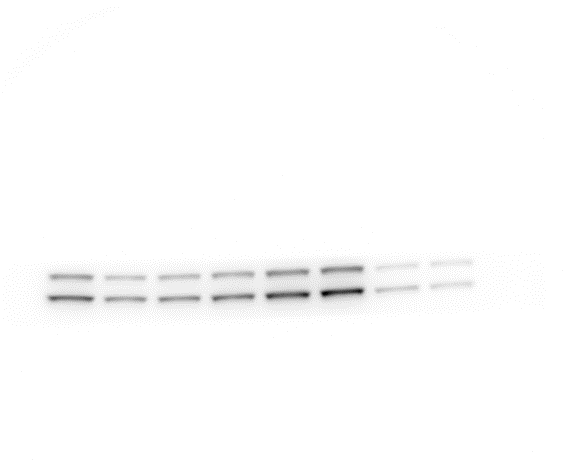


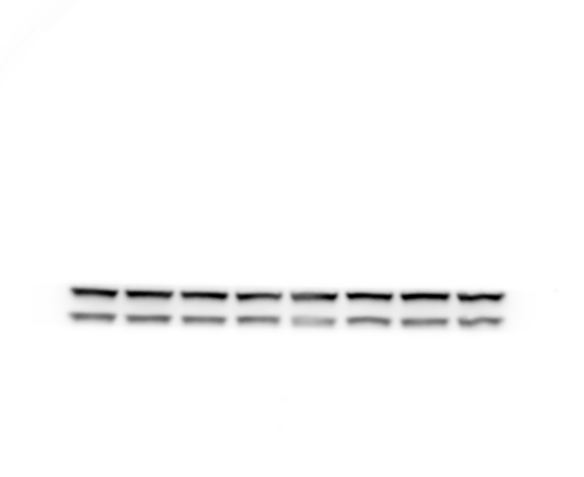


Figure 2A p38 Figure 2A p-p38


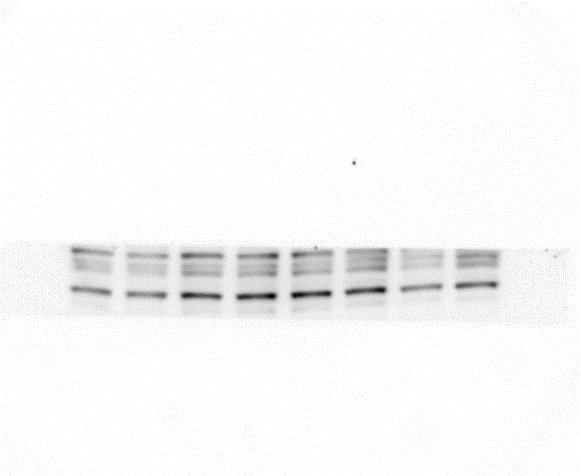


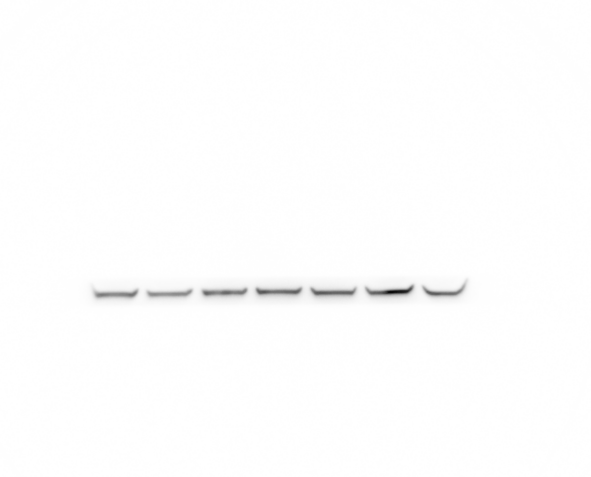


Figure 2A α-Tubulin Figure 2G α-Tubulin


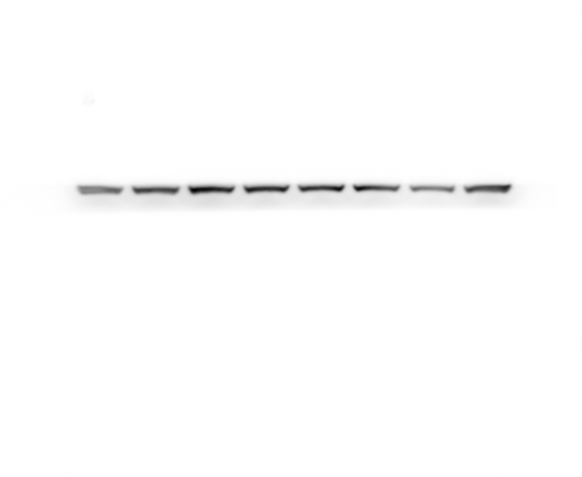

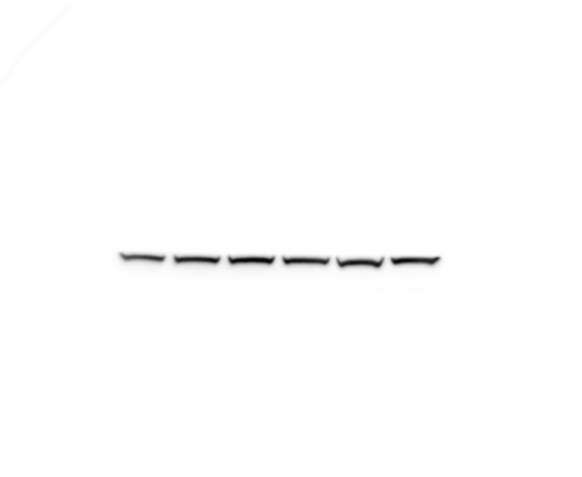


Figure 2G ERK Figure 2G p-ERK


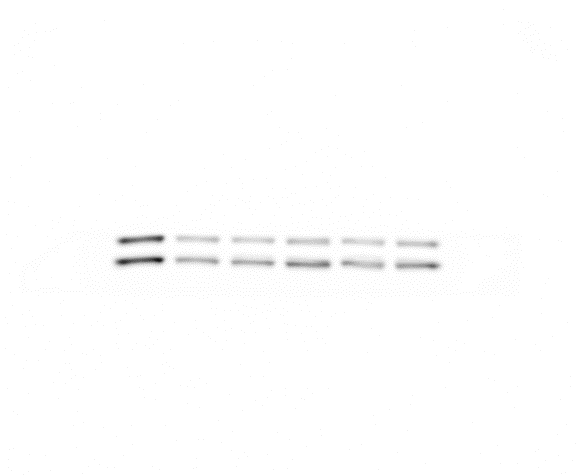


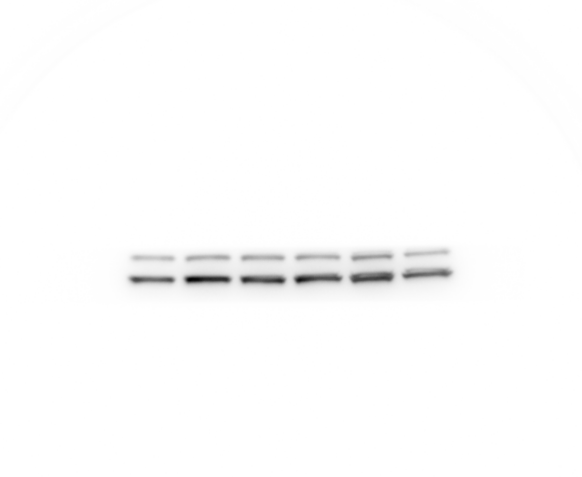


Figure 2G JNK Figure 2G p-JNK


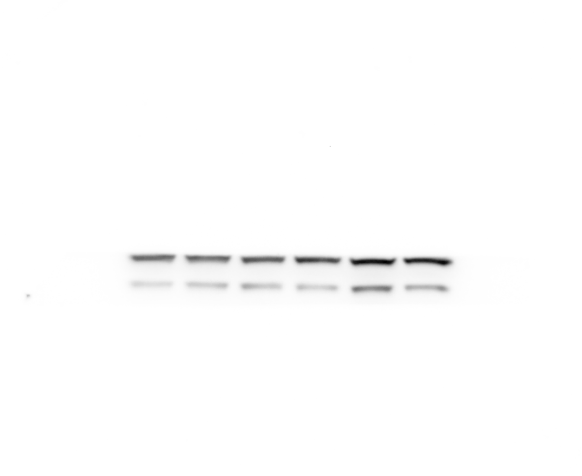


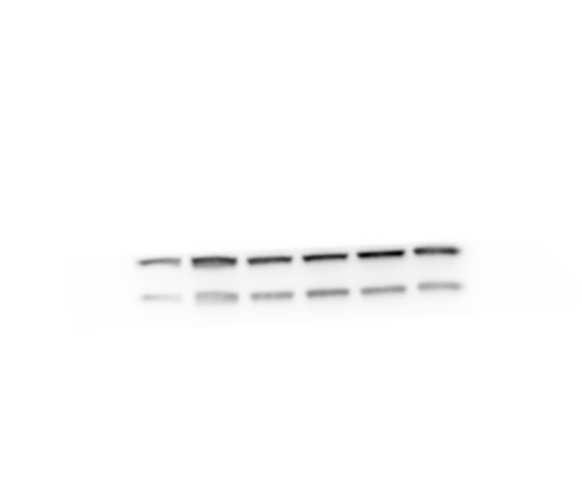


Figure 2G p38 Figure 2G p-p38


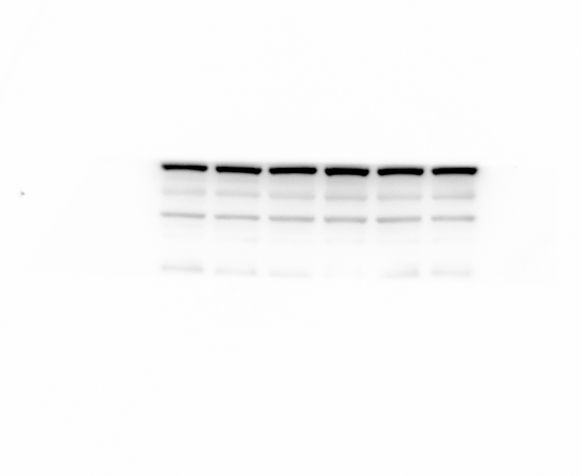

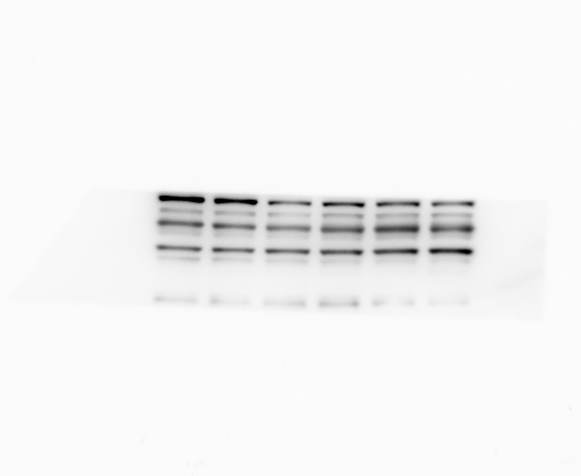


Figure 3A AMPKα Figure 3A p-AMPKα


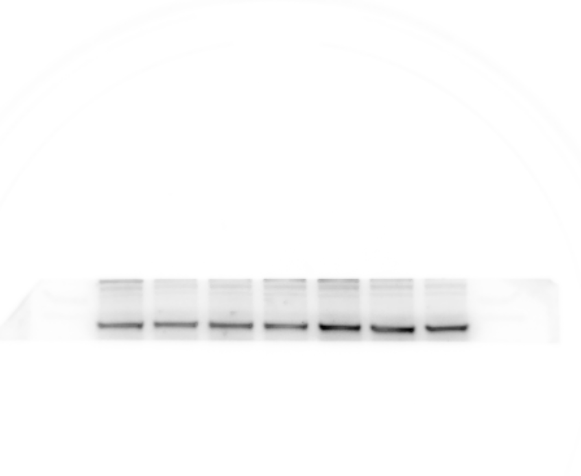

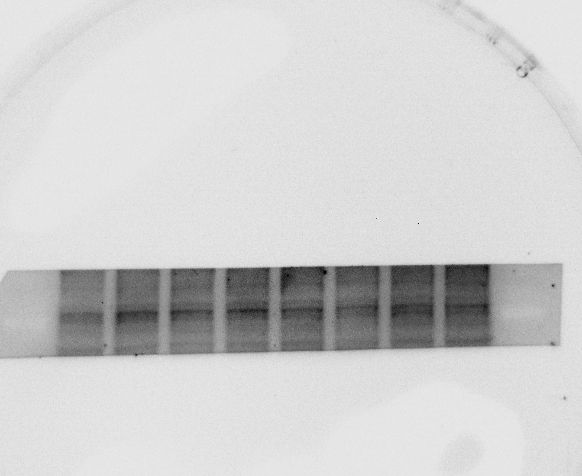


Figure 3A ACC Figure 3A p-ACC


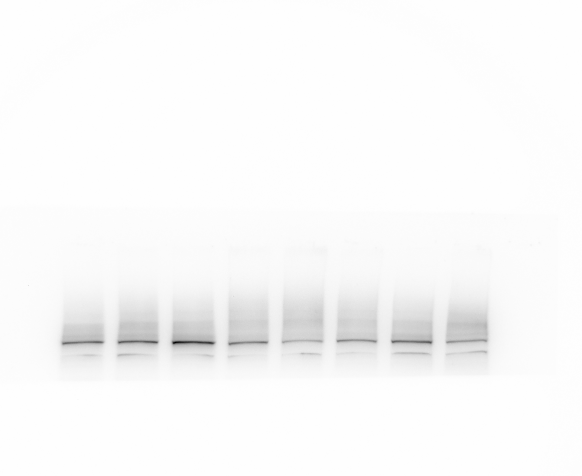

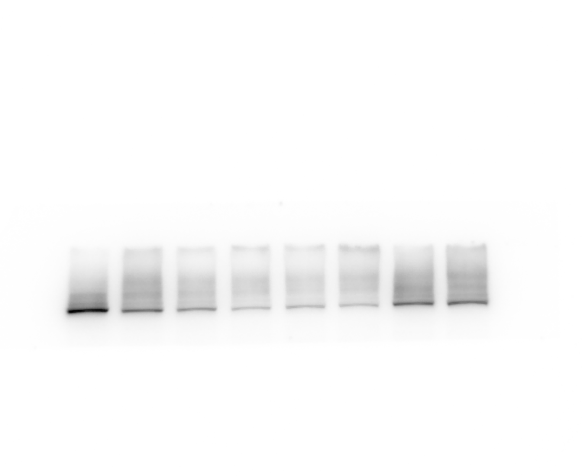


Figure 3A α-Tubulin Figure 3F α-Tubulin


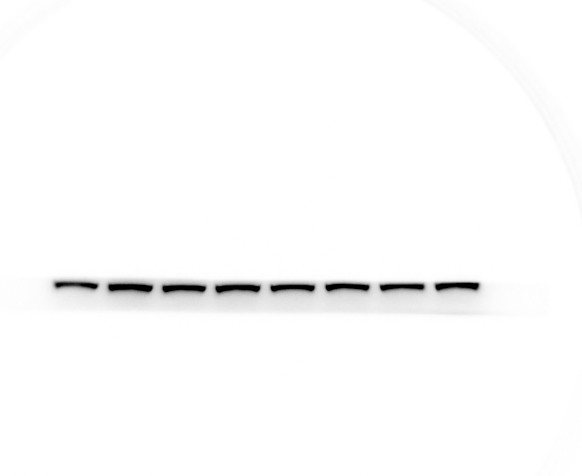

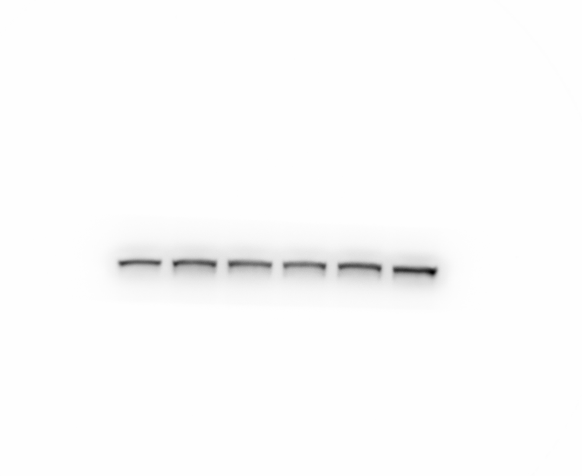


Figure 3F AMPKα Figure 3F p-AMPKα


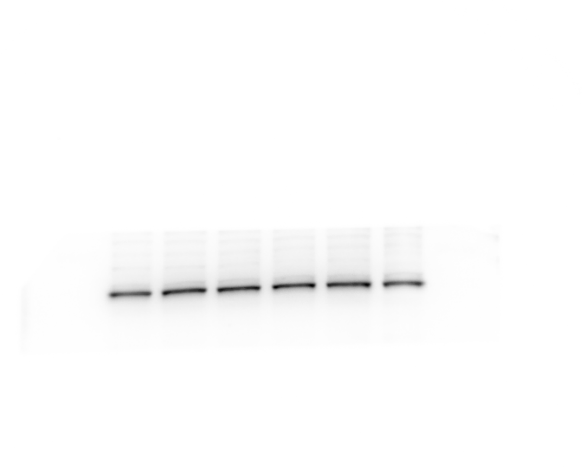

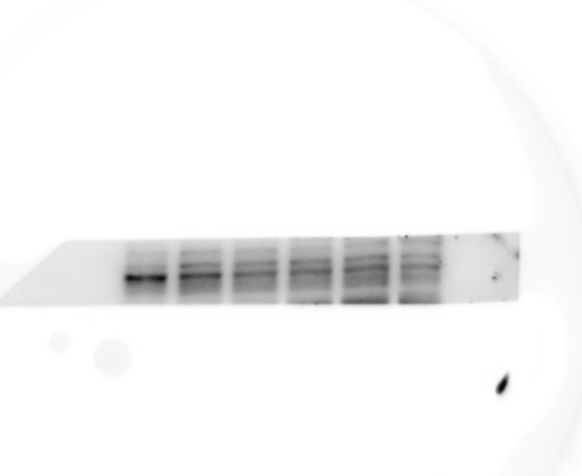


Figure 3F ACC Figure 3F p-ACC


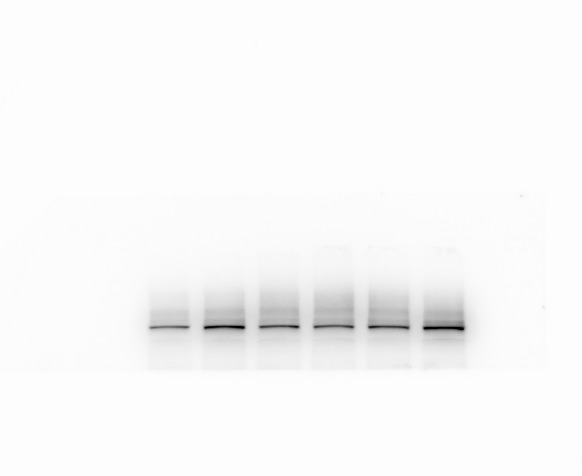

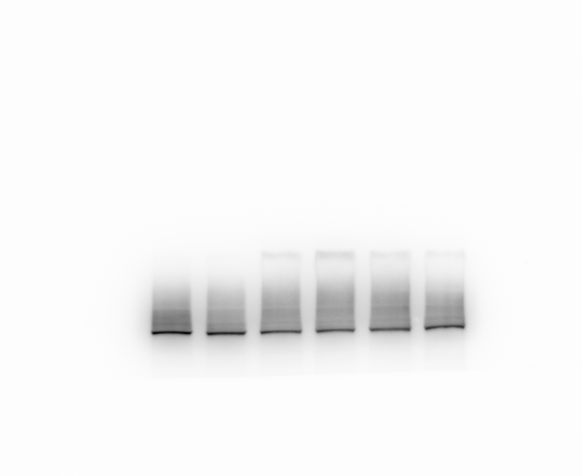


Figure 4A p65 Figure 4A p-p65


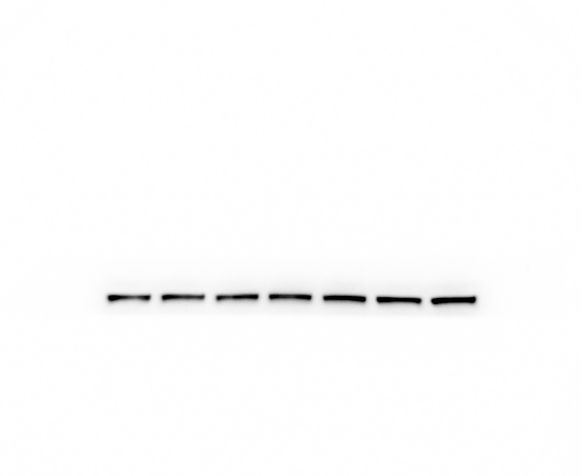


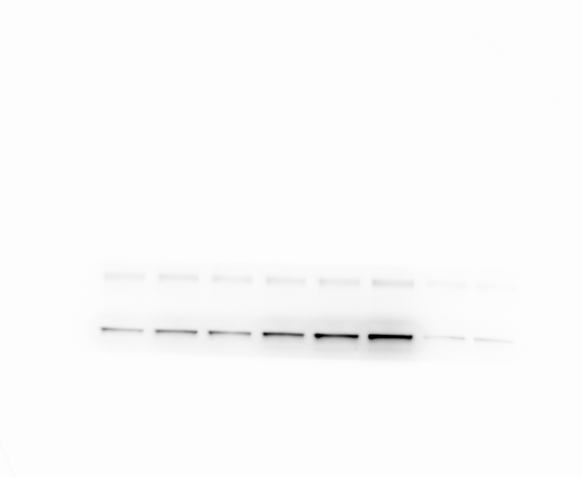


Figure 4A IκBα Figure 4A p-IκBα


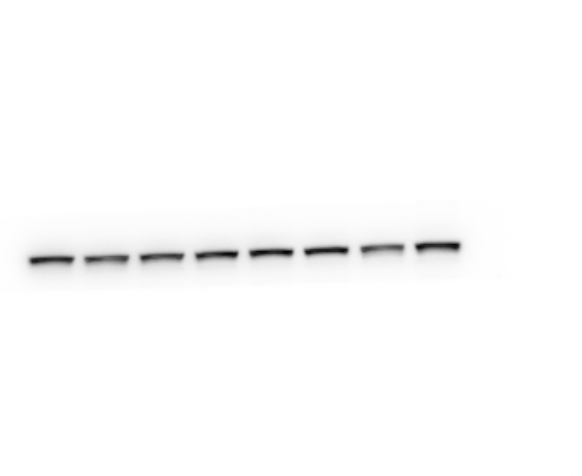

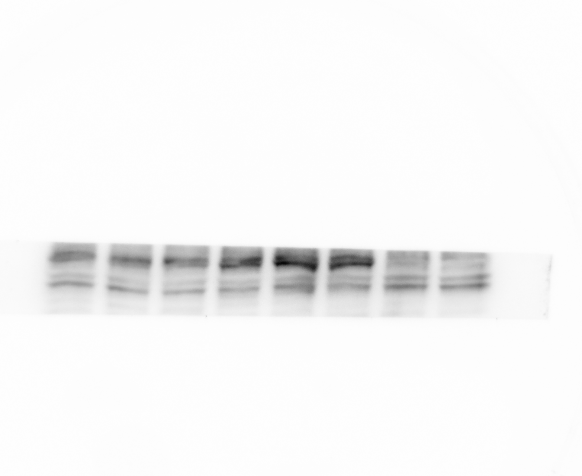


Figure 4A α-Tubulin Figure 4F α- Tubulin


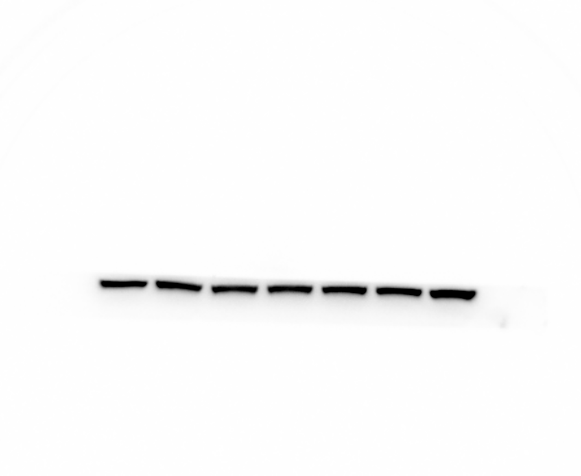

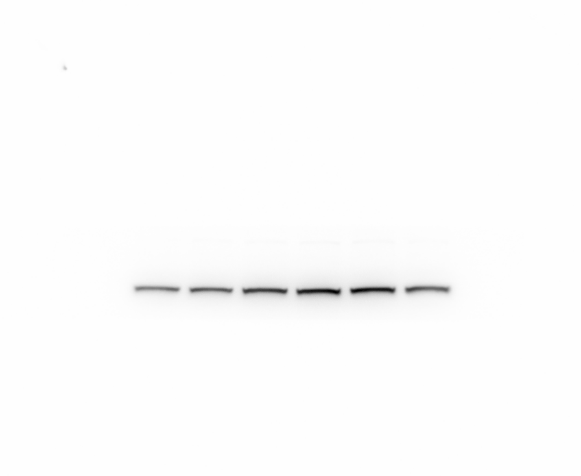


Figure 4F p65 Figure 4F p-p65


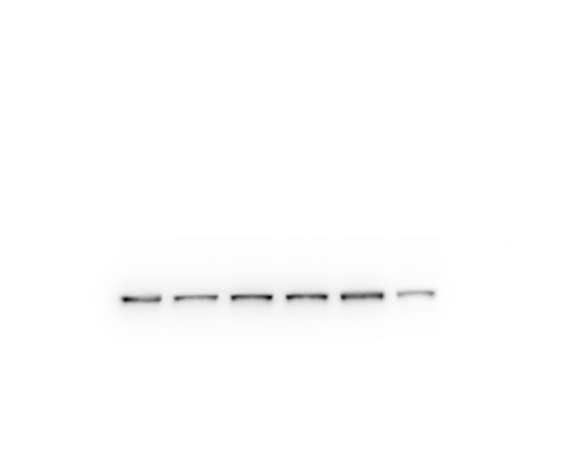

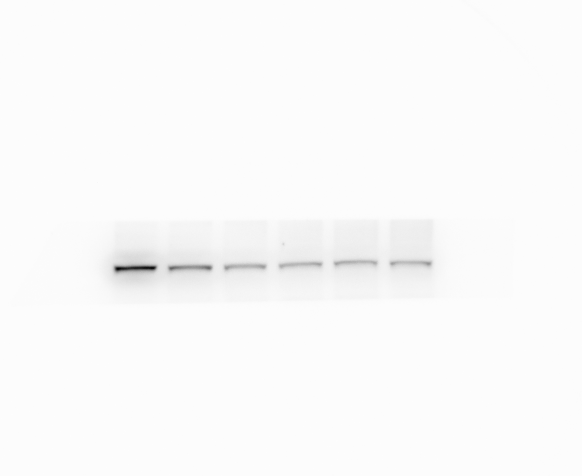


Figure 4F IκBα Figure 4F p-IκBα


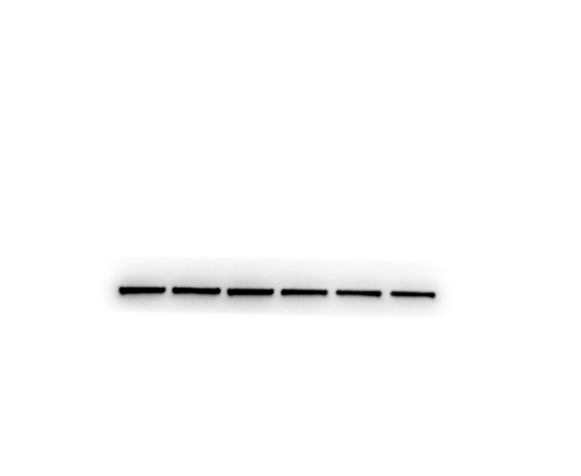

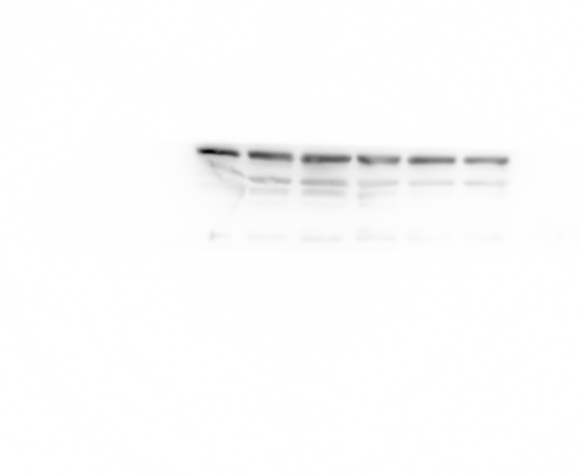


Figure 5A RSK Figure 5A p-RSK


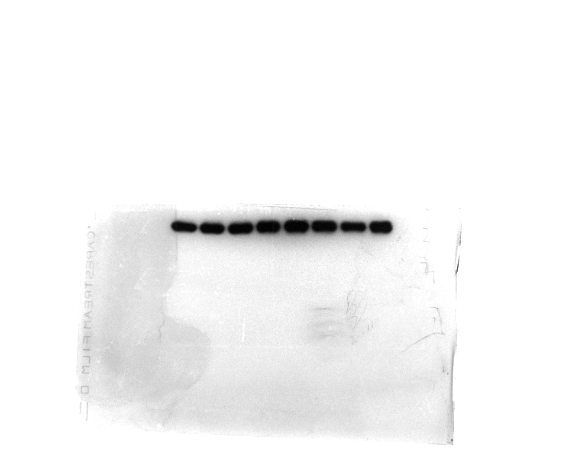

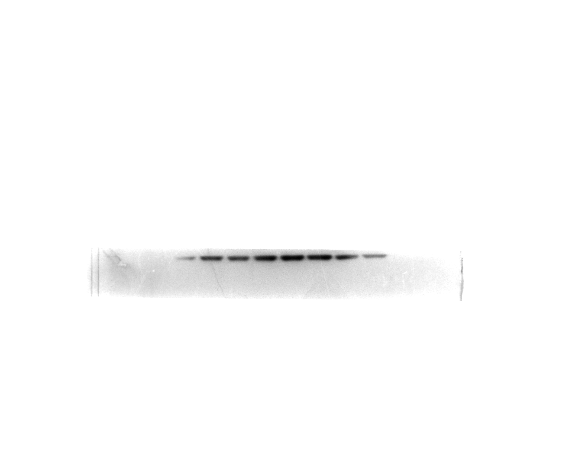


Figure 5A MSK Figure 5A p-MSK


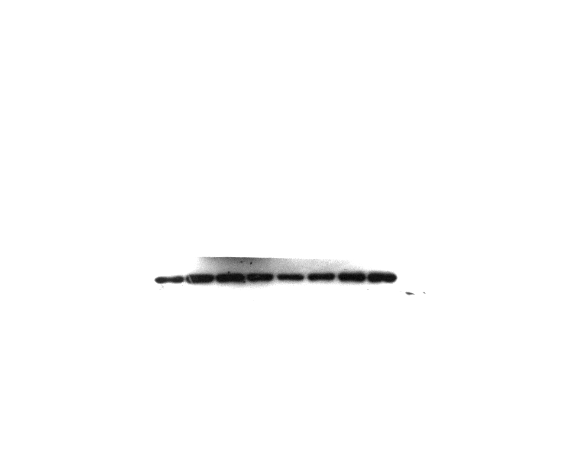

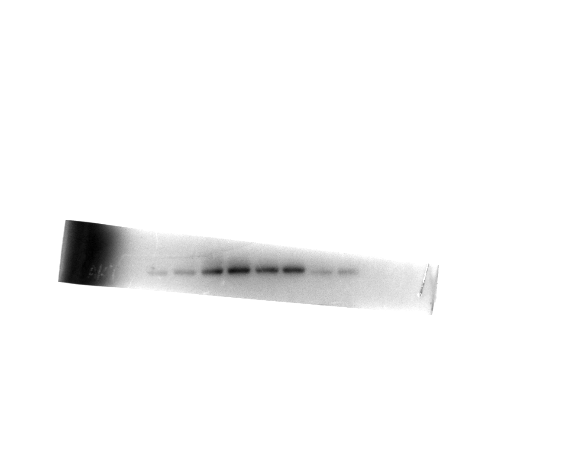


Figure 5A β-actin Figure 7A α-Tubulin


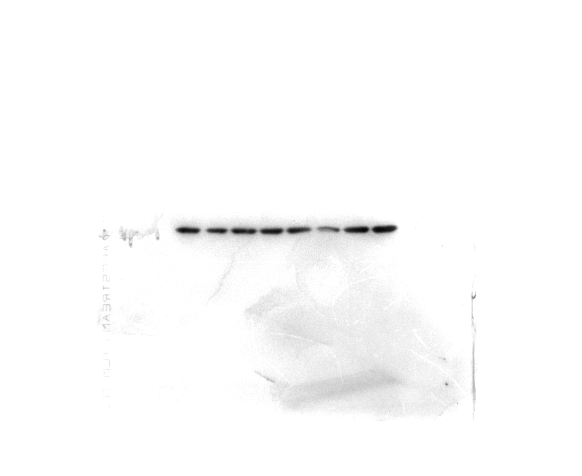


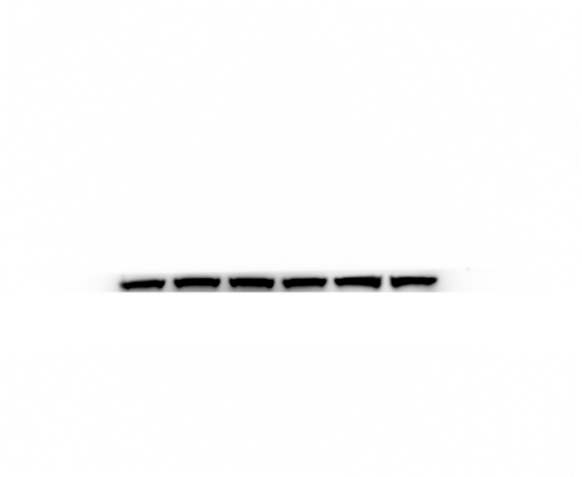


Figure 7A ERK Figure 7A p-ERK


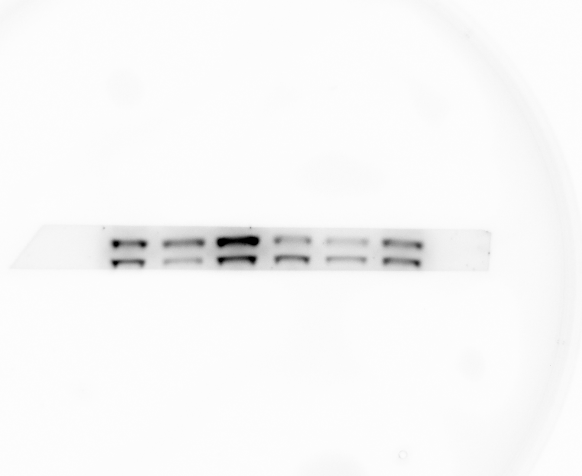


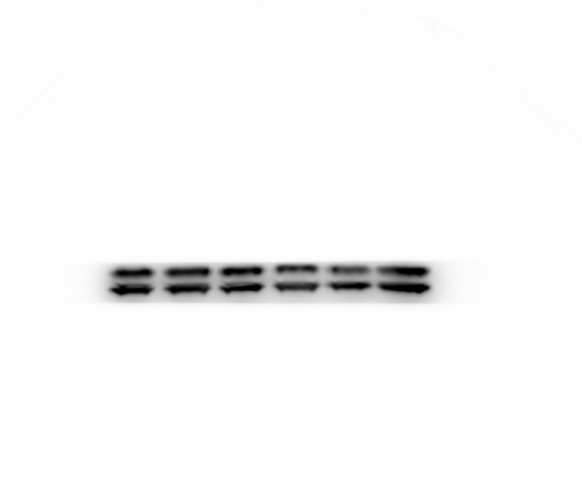


Figure 7A JNK Figure 7A p-JNK


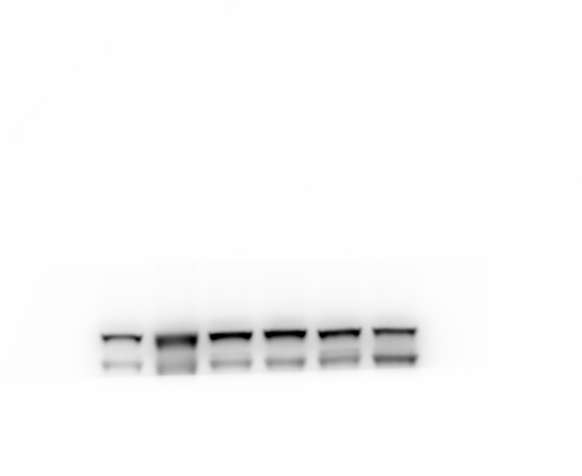

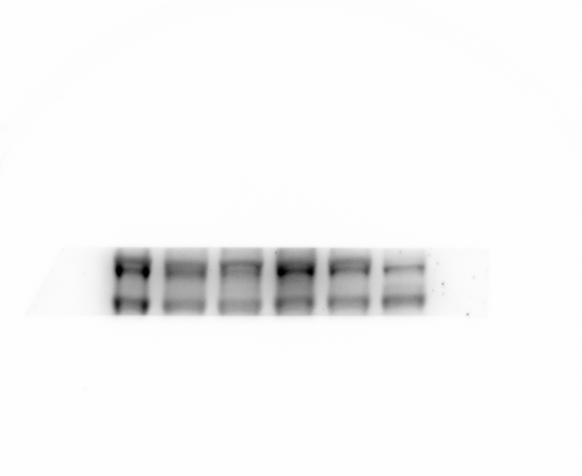


Figure 7A p38 Figure 7A p-p38


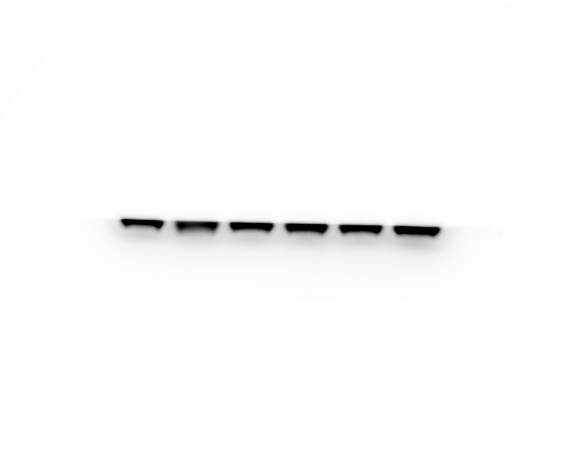

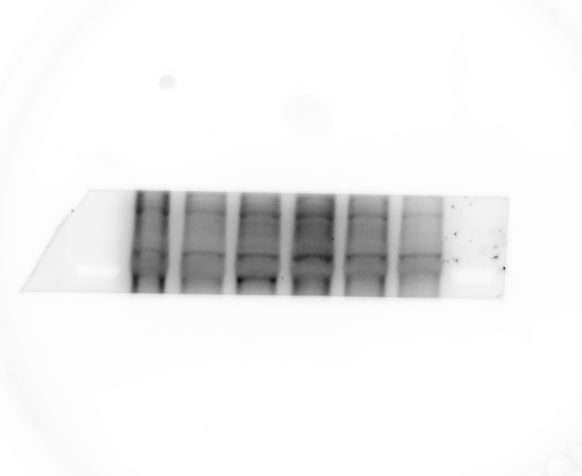


Figure 8A p65 Figure 8A p-p65


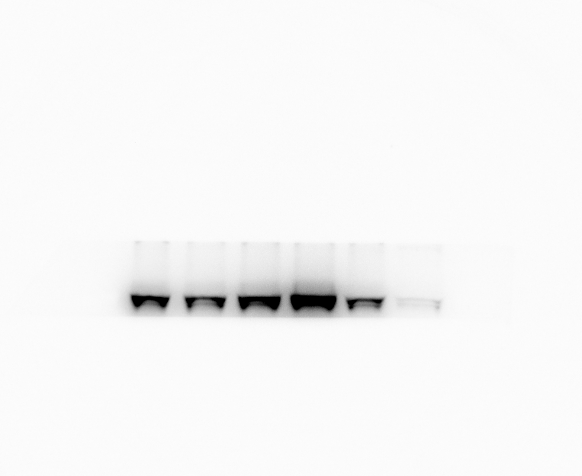

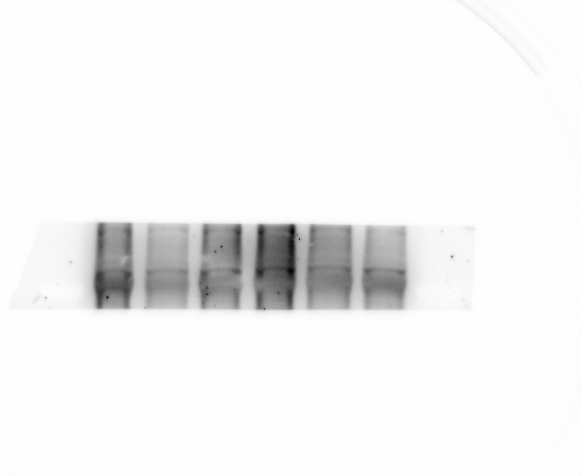


Figure 8A IκBα Figure 8A p-IκBα


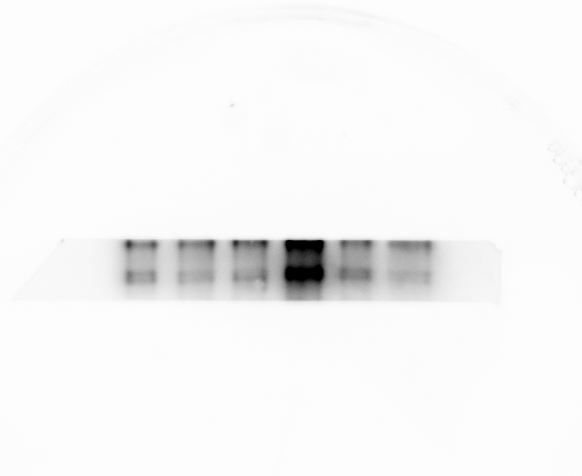

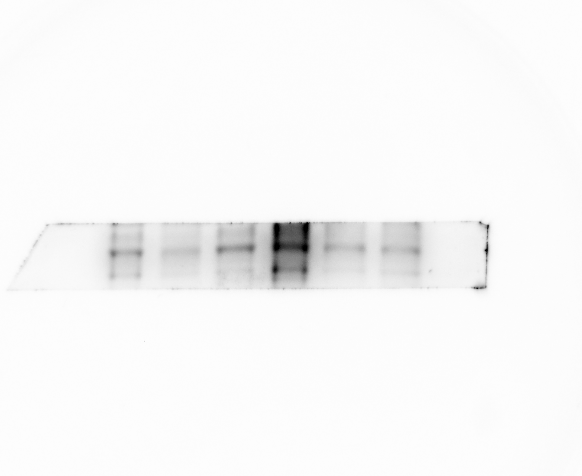


Figure 8G MPO Figure 8G β-actin


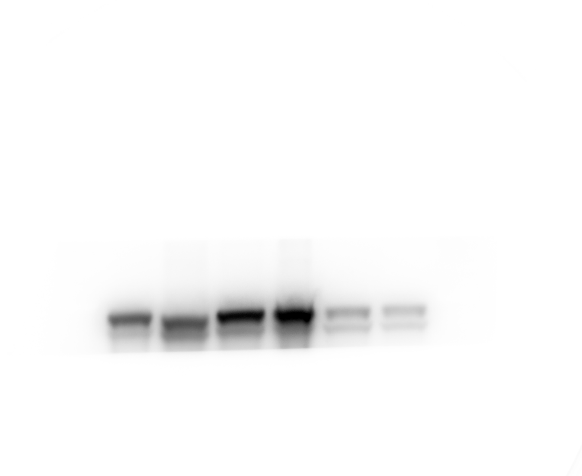

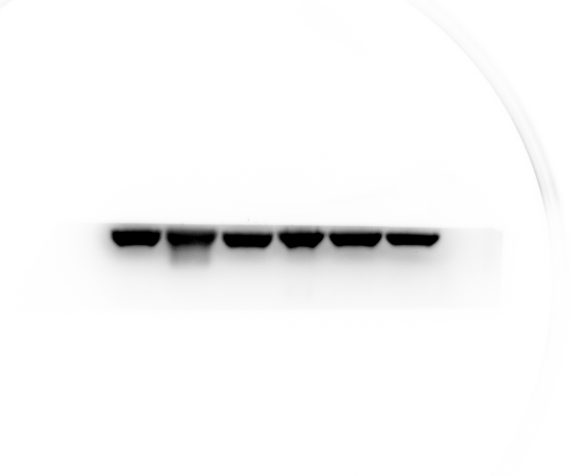


Figure 9A AMPKα Figure 9A p-AMPKα


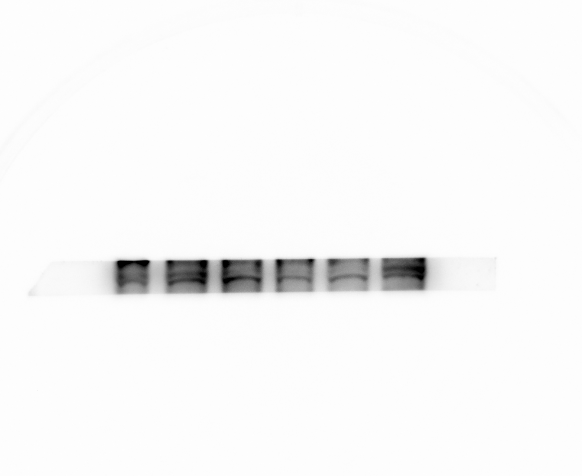

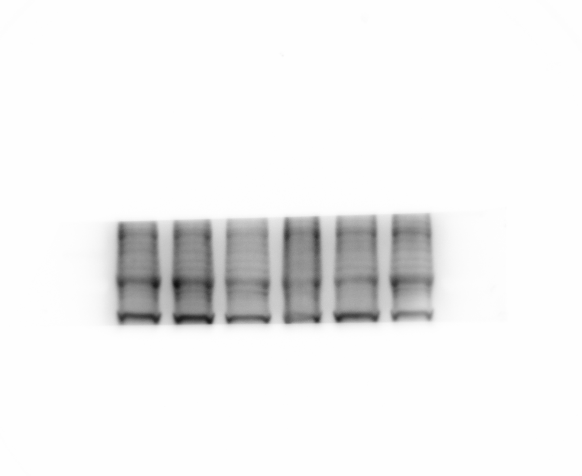


Figure 9A ACC Figure 9A p-ACC


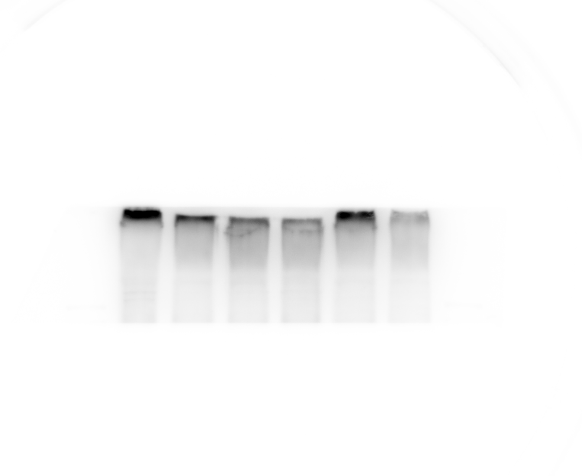

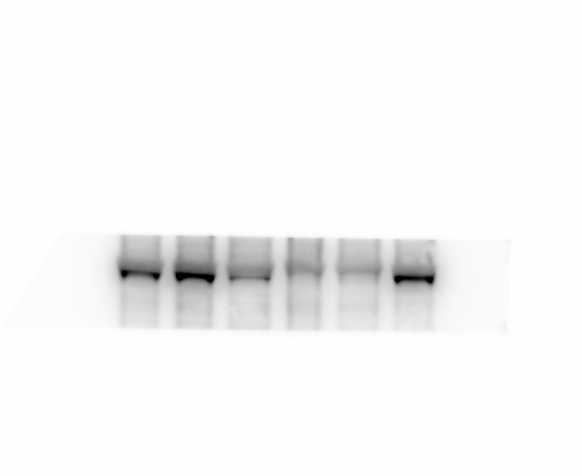

Supplement: Supplementary file 1 — Supplementary Information [file 41598_2020_58566_MOESM1_ESM.docx]
